# Natural Selection Signatures in the Hondo and Ryukyu Japanese Subpopulations

**DOI:** 10.1093/molbev/msad231

**Published:** 2023-10-30

**Authors:** Xiaoxi Liu, Masatoshi Matsunami, Momoko Horikoshi, Shuji Ito, Yuki Ishikawa, Kunihiko Suzuki, Yukihide Momozawa, Shumpei Niida, Ryosuke Kimura, Kouichi Ozaki, Shiro Maeda, Minako Imamura, Chikashi Terao

**Affiliations:** Laboratory for Statistical and Translational Genetics, RIKEN Center for Integrative Medical Sciences, Yokohama, Japan; Clinical Research Center, Shizuoka General Hospital, Shizuoka, Japan; Department of Advanced Genomic and Laboratory Medicine, Graduate School of Medicine, University of the Ryukyus, Nishihara-Cho, Japan; Laboratory for Genomics of Diabetes and Metabolism, RIKEN Center for Integrative Medical Sciences, Yokohama, Japan; Laboratory for Statistical and Translational Genetics, RIKEN Center for Integrative Medical Sciences, Yokohama, Japan; Laboratory for Statistical and Translational Genetics, RIKEN Center for Integrative Medical Sciences, Yokohama, Japan; Laboratory for Genotyping Development, RIKEN Center for Integrative Medical Sciences, Yokohama, Japan; Laboratory for Genotyping Development, RIKEN Center for Integrative Medical Sciences, Yokohama, Japan; Core Facility Administration, Research Institute, National Center for Geriatrics and Gerontology, Obu, Japan; Department of Human Biology and Anatomy, Graduate School of Medicine, University of the Ryukyus, Nishihara-Cho, Japan; Medical Genome Center, Research Institute, National Center for Geriatrics and Gerontology, Obu, Japan; Department of Advanced Genomic and Laboratory Medicine, Graduate School of Medicine, University of the Ryukyus, Nishihara-Cho, Japan; Division of Clinical Laboratory and Blood Transfusion, University of the Ryukyus Hospital, Okinawa, Japan; Department of Advanced Genomic and Laboratory Medicine, Graduate School of Medicine, University of the Ryukyus, Nishihara-Cho, Japan; Division of Clinical Laboratory and Blood Transfusion, University of the Ryukyus Hospital, Okinawa, Japan; Laboratory for Statistical and Translational Genetics, RIKEN Center for Integrative Medical Sciences, Yokohama, Japan; Clinical Research Center, Shizuoka General Hospital, Shizuoka, Japan; The Department of Applied Genetics, School of Pharmaceutical Sciences, University of Shizuoka, Shizuoka, Japan

**Keywords:** natural selection, Japanese, Hondo, Ryukyu, iHS, FastSMC, *ALDH2*

## Abstract

Natural selection signatures across Japanese subpopulations are under-explored. Here we conducted genome-wide selection scans with 622,926 single nucleotide polymorphisms for 20,366 Japanese individuals, who were recruited from the main-islands of Japanese Archipelago (Hondo) and the Ryukyu Archipelago (Ryukyu), representing two major Japanese subpopulations. The integrated haplotype score (iHS) analysis identified several signals in one or both subpopulations. We found a novel candidate locus at *IKZF2,* especially in Ryukyu. Significant signals were observed in the major histocompatibility complex region in both subpopulations. The lead variants differed and demonstrated substantial allele frequency differences between Hondo and Ryukyu. The lead variant in Hondo tags HLA-A*33:03-C*14:03-B*44:03-DRB1*13:02-DQB1*06:04-DPB1*04:01, a haplotype specific to Japanese and Korean. While in Ryukyu, the lead variant tags DRB1*15:01-DQB1*06:02, which had been recognized as a genetic risk factor for narcolepsy. In contrast, it is reported to confer protective effects against type 1 diabetes and human T lymphotropic virus type 1-associated myelopathy/tropical spastic paraparesis. The FastSMC analysis identified 8 loci potentially affected by selection within the past 20–150 generations, including 2 novel candidate loci. The analysis also showed differences in selection patterns of *ALDH2* between Hondo and Ryukyu, a gene recognized to be specifically targeted by selection in East Asian. In summary, our study provided insights into the selection signatures within the Japanese and nominated potential sources of selection pressure.

## Introduction

Approximately 100,000 yr ago, anatomically modern human populations started moving out of Africa and initiated a global migration (Klein 2008). The novel environments, which might be markedly different from their African origin, are believed to have driven selection for new traits important for human survival, and have left discernible signatures within the human genome. With the advent of high-throughput genotyping technology and analytical methodologies, dozens of genetic loci have been linked to the natural selection (Benton et al. 2021). These loci reflect adaptations to various challenges such as high-altitude (Yang et al. 2017), novel pathogens (Kwiatkowski 2005), changes of food sources (Mathieson and Mathieson 2018), etc. Detection of positive selection for each local population not only greatly enhances our understanding of the adaptive evolution, but also has important medical implications (Vasseur and Quintana-Murci 2013).

In the Japanese population, genetic loci harboring *ALDH2* (Oota et al. 2004), *EDAR* (Fujimoto et al. 2008; Kimura et al. 2009), and major histocompatibility complex (*MHC*) region (Kawashima et al. 2012) were reported to be under positive selection based on the candidate gene approach. By applying the singleton density score method to 2,234 Japanese whole-genome sequencing (WGS) data, a genome-wide scan of very recent selection signature detected four loci including the ADH cluster, MHC region, *BRAP-ALDH2*, and *SERHL2* (Okada et al. 2018). A recent study using large-scale microarray data of 170,882 subjects from the Biobank Japan (BBJ) reported 29 candidate loci by the ascertained sequentially Markovian coalescent (ASMC) method, and two loci including ADH cluster and MHC by the integrated haplotype score (iHS) method, which remarkably expanded the number of candidate genes subjected to natural selection (Yasumizu et al. 2020). In the context of recent advances, several critical issues have not been adequately addressed yet. First, the principal component analysis (PCA) demonstrated a dual population structure in the Japanese: the Hondo (literally translated as main-islands) cluster on the Japanese Archipelago and the Ryukyu cluster on the Ryukyu Archipelago (Yamaguchi-Kabata et al. 2008; Sakaue et al. 2020). The different peopling histories of Hondo and Ryukyu populations, leading to varying levels of admixture between neolithic Jomon and Yayoi ancestral groups, have been proposed to underlie this unique population structure (Japanese Archipelago Human Population Genetics Consortium 2012; Bendjilali et al. 2014; Koganebuchi and Kimura 2019) (Supplementary Notes S1 and S2). Genetic differentiation was observed among island groups of the Ryukyu Archipelago, and there is little genetic affinity between aboriginal Taiwanese and any of the Ryukyu people despite the geographical proximity (Sato et al. 2014). In the essential context of Japan's dual population structure, it is worth noting that previous studies, though not primarily focused on the Japanese population but including it in their scope, have predominantly relied on individuals recruited from Hondo (Voight et al. 2006; Sabeti et al. 2007; Johnson and Voight 2018). Moreover, those studies that do focus on the Japanese did not distinguish adequately between Hondo and Ryukyu (Okada et al. 2018; Yasumizu et al. 2020). Large-scale analyses specifically targeting the Ryukyu subpopulation are notably absent (Liu et al. 2017). This represents a substantial knowledge gap in our understanding of natural selection within the Japanese population. Second, there is geographic variation in disease prevalence, pathogen subtype, and infection rate of pathogens between Hondo and Ryukyu (Hayashi et al. 1990; Fukiyama et al. 2000; Aoki et al. 2008; Takeuchi et al. 2014). For example, strains of *Helicobacter pylori*, which are associated with the development of gastric cancer, differ between Hondo and Ryukyu; the strains specific to Ryukyu are less virulent (Suzuki et al. 2022). Another example is a notably higher prevalence of Human T lymphotropic virus type 1 (HTLV-1) infection in Ryukyu than in Hondo, which can result in a range of clinical manifestations (Morikawa et al. 1988; Watanabe 2011; Iwanaga 2020). It would be intriguing to examine whether there are any differences in selection profiles between Hondo and Ryukyu, for which one possible explanation is provided by varying environmental factors. Third, replication studies to confirm the previously reported signals remain much lacking. Finally, studies with DNA microarrays designed to contain more East Asian-specific probes hold the promise to capture novel signals and may further advance our understanding of the natural selection signatures in the Japanese (Kawai et al. 2015).

To address the above issues, we carried out genome-wide selection scans using data of 20,366 individuals who were recruited from both Hondo and Ryukyu and had been genotyped on the Illumina Infinium Asian Screening Array (ASA), an array specifically designed to contain East Asian-specific variants. In order to make our results comparable with the previous study (Yasumizu et al. 2020), two methods: iHS (Voight et al. 2006) and FastSMC (Nait Saada et al. 2020) were applied. The iHS detects recent and ongoing selection signals, such as soft sweep based on phased haplotype information. Additionally, FastSMC offers an alternative approach for identifying candidate regions potentially targeted by selection through the inference of identity-by-descent (IBD) sharing (Palamara et al. 2018; Nait Saada et al. 2020).

## Results

### Fine-scale Genetic Structure of the Japanese Population, Especially the Ryukyu Cluster

A total of 20,366 individuals from two cohorts in Japan were analyzed in this study. The first cohort consisted of 13,753 participants at the National Center for Geriatrics and Gerontology (NCGG) Biobank in Japan (Shigemizu et al. 2021). The second cohort consisted of 6,613 participants who were recruited at Okinawa Prefecture through the Okinawa Bioinformation Bank (OBi) Project in which detailed geographical information of origins (including islands in Okinawa Prefecture) are available in some participants (Matsunami et al. 2021) (Supplementary Table S1; Supplementary Note S2). We merged the two cohorts and a total of 622,926 SNPs were available for analysis. For quality control (QC) (see Methods), we first removed 65,826 variants that have a call rate less than 97% or a Hardy–Weinberg equilibrium *P*-value (HWE-P) < 1 × 10^−6^. Then we removed 745 samples with a sample call rate of less than 97%. Furthermore, we excluded 1,585 closely related individuals with a shared IBD (*π^^^*) >=0.25. Based on the PCA, we additionally removed 45 samples whose PC1 or PC2 showed 3 standard deviations (SD) from the mean PC values, 25 samples overlapping with the Chinese cluster, and 67 samples overlapping with the Korean cluster. Because deviations from HWE can occur when examining data comprising distinct subpopulations, we conducted additional analyses after the PCA–UMAP (Uniform Manifold Approximation and Projection) (shown in a later section). We recalculated HWE for SNPs that failed the QC, separately for each subgroup. Through this approach, we were able to recover 3,033 variants that only significantly deviated from HWE when the whole dataset was considered. The final dataset consisted of 17,932 participants with 560,133 variants with an average SNP call rate of 99.85%.

**Fig. 1.**
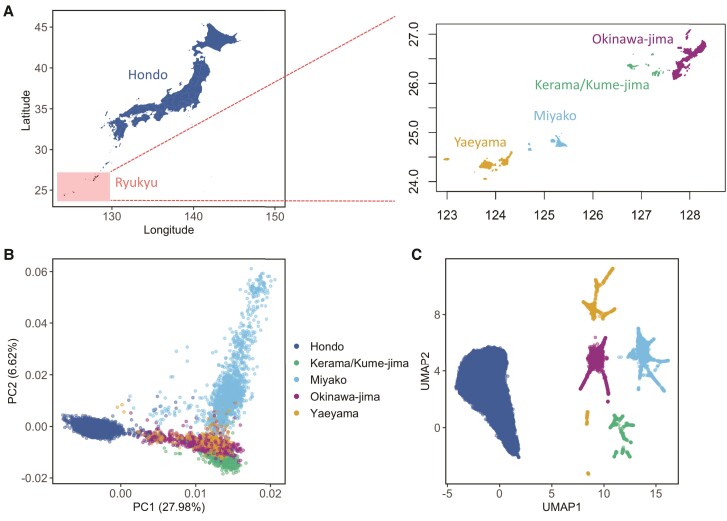
Fine-scale population structure of the Japanese population. a) The geography of the Japanese archipelago and the Ryukyu archipelago. On the right, a zoomed-in view of the Ryukyu archipelago is displayed, consisting of four major island groups: Okinawa-jima, Kerama/kume-jima, Miyako, and Yaeyama. b) The PCA plot of 17,932 Japanese samples. c) The PCA-UMAP plot of 17,932 Japanese samples. The geographic region associated with each PCA-UMAP cluster were inferred based on individuals with clear ancestry records (refer to Supplementary Table S2). Samples located within the same PCA-UMAP cluster were regarded as originating from the same region. Note that the colors assigned to the samples in the PCA plot (panel b) were consistent with the colors assigned to the samples in the PCA-UMAP plot (panel c) to ensure coherence and clarity.

Mirroring the geography of Japan, PCA based on 163,727 pruned tag SNPs separated our study samples into the Hondo and Ryukyu clusters (Fig. 1a and b), which was in alignment with previous studies (Yamaguchi-Kabata et al. 2008; Sakaue et al. 2020). The Hondo cluster lies in-between Chinese/Korean and Ryukyu clusters (Supplementary Fig. S1A), suggesting that selection signals in the Ryukyu cluster cannot be inferred by using only the Hondo cluster (and Chinese/Korean cluster). To validate that the PCA results were not influenced by technical batch effects because the genotyping was conducted independently from two cohorts, we performed PCA using only the OBi cohort (which had a sufficient number of samples from both Ryukyu and Hondo) and confirmed the projections with NCGG samples. The majority of NCGG samples were found in the expected Hondo cluster, indicating that the PCA results accurately reflect the population structure (Supplementary Fig. S2).

The PCA-UMAP analysis revealed a high-resolution fine-scale population structure in the Japanese, in which mainly five distinct clusters were identified (Fig. 1c) in addition to clear separation of Japanese subjects from other East Asian populations (Supplementary Fig. S1B). The NCGG samples mostly fell into a single cluster representing the Hondo cluster (Supplementary Table S1). The OBi subjects scattered across the other four clusters and we took advantage of 2,671 individuals with clear records of ancestry (all four grandparents born in the same island group) to infer the composition of non-Hondo clusters (Supplementary Table S2). We observed that these clusters correspond well to four major Ryukyu island groups, namely Okinawa, Yaeyama, Miyako, and Kerama/Kume-jima Islands. The geographic regions inferred from PCA-UMAP matched the birth records of 2,106 out of 2,195 samples (95.94%) in non-Hondo clusters and 475 out of 476 OBi samples (99.80%) in the Hondo cluster (Supplementary Table S2). Based on the clusters of PCA-UMAP, we redefined samples into five subpopulations: Hondo (*N* = 13,533), Okinawa-jima (*N* = 1,747), Miyako (*N* = 1,406), Yaeyama (*N* = 807), Kerama/Kume-jima (*N* = 439) (Supplementary Table S1).

### Genetic Distance Inferred by the *F_ST_* Analysis

We considered genetic distance using Hudson's fixation index (*F_ST_*) for pairs of the five subpopulations (Table 1). Hondo showed larger *F_ST_* values with each Ryukyu subpopulation than those among Ryukyu subpopulations, which is consistent with the PCA analysis. Miyako and Kerama/Kume-jima (*F_ST_* ± standard error = 9.84 × 10^−4^ ± 3.53 × 10^−6^) showed the highest *F_ST_* value among the Ryukyu subpopulations. We computed *P*-values of *F_ST_* for 415,141 variants which have a minor allele frequency (MAF) >= 1% in the combined dataset to characterize the genetic distance. We identified a genome-wide significant peak in the MHC region (*P* < 1.20 × 10^−7^, 0.05/415,141) with rs2071653 as the leading variant (*F_ST_* = 0.168, *P* = 1.05 × 10^−7^) (Supplementary Fig. S3). Although no variant outside the MHC reached genome-wide significance after multiple testing adjustments, we scrutinized the nonsynonymous variants showing the highest *F_ST_* values between the Hondo and Ryukyu. Notably, rs671 in *ALDH2* ranked at the top (*F_ST_* = 0.088, *P* = 1.16 × 10^−4^) (Supplementary Table S3). The derived allele of rs671 (p.Glu504Lys) has been known to reduce enzyme activity of ALDH2 (Matsuo et al. 2006), leading to the alcoholic intolerance, and has significantly higher allele frequency (AF) in Hondo compared with Ryukyu (0.29 vs. 0.11, *P* = 4.80 × 10^−236^, Chi-squared test).

**Table 1:** Pair-wise *F_ST_* of Japanese subpopulations defined by PCA-UMAP analysis

Population 1	Population 2	*F_ST_*	SE
Hondo	Okinawa-jima	2.75E−03	6.73E−06
Hondo	Miyako	3.35E−03	7.97E−06
Hondo	Yaeyama	2.95E−03	7.38E−06
Hondo	Kerama/Kume-jima	3.49E−03	8.72E−06
Okinawa-jima	Miyako	4.05E−04	1.49E−06
Okinawa-jima	Yaeyama	2.53E−04	1.55E−06
Okinawa-jima	Kerama/Kume-jima	4.77E−04	2.39E−06
Miyako	Yaeyama	4.99E−04	2.12E−06
Miyako	Kerama/Kume-jima	9.84E−04	3.53E−06
Yaeyama	Kerama/Kume-jima	7.61E−04	3.43E−06

SE, stand error.

### Shared Candidate Loci Influenced by Selection in Hondo and Ryukyu Subpopulations

**Fig. 2.**
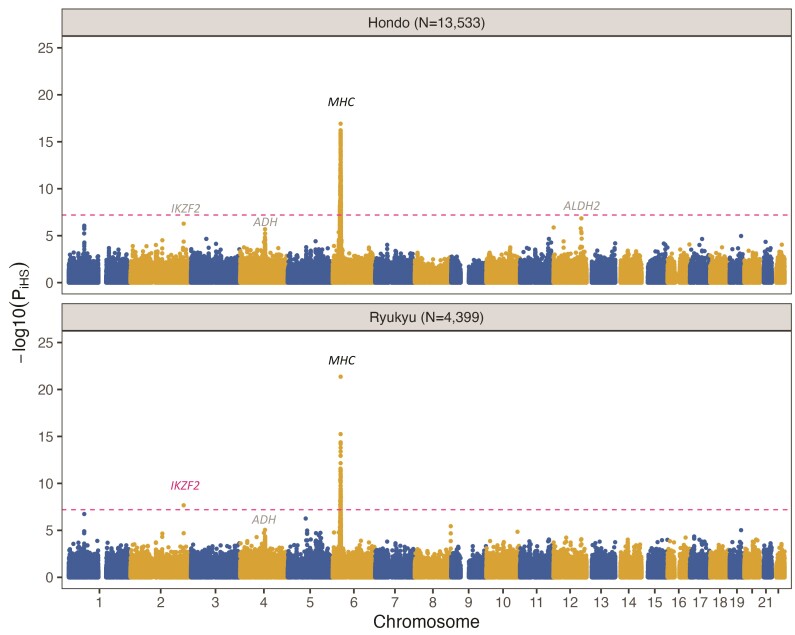
Genetic loci under positive selection in the Japanese population based on iHS analysis in Hondo (top) and Ryukyu (bottom). The –log10(*P_iHS_*) value (*y* axis) and the chromosomal position (*x* axis) of each SNP are plotted across the genome. The red dashed line indicates the Bonferroni-corrected genome-wide significance threshold (*P* < 6.33 × 10^−8^). Candidate genes previously reported to be involved in positive selection in the Japanese population are colored in black, while novel genes are marked in red. We have also identified subpeaks, including *ADH*, *ALDH2* (in Hondo only), and *IKZF2* (in Hondo only), where the *P_iHS_* value slightly falls short of the genome-wide significance. These subpeaks are represented in grey.

We conducted iHS analysis in Hondo and Ryukyu separately and detected two candidate genetic loci potentially affected by selection at the genome-wide significance (*P_iHS_* < 6.33 × 10^−8^, 0.05/394,906/2, see Methods), including the MHC region and *IKZF2* (Fig. 2 and Table 2). The Quantile-Quantile (QQ) plots indicate there is no systematic bias stratified across different frequency bins (Supplementary Fig. S4). We confirmed that both loci do not overlap with any known structural variants (SVs) (see Methods). A signal from MHC (6p22) was observed in both Hondo and Ryukyu. The signal from *IKZF2* (2q34) in Ryukyu (*P_iHS_* = 2.15 × 10^−8^) slightly fall short of the genome-wide significance in Hondo (*P_iHS_* = 5.25 × 10^−7^, Table 2). We also noticed subpeaks including *ALDH2* (12q24.12), which had rs671 as the lead variant (*P_iHS_* = 1.43 × 10^−7^ in Hondo) and *ADH* (4q23). In comparison with the previous iHS analysis based on BBJ samples (Yasumizu et al. 2020), we could replicate all two reported loci: MHC and *ADH*, while the significance in *IKZF2* and the suggestive signal in *ALDH2* were newly identified in this study. We also performed iHS for each Ryukyu subpopulation and found generally consistent results with those in the entire Ryukyu population (Supplementary Fig. S5).

**Table 2:** Lead variants of candidate significant loci in Hondo and Ryukyu uncovered by iHS analysis

CHR	SNP	BP	Gene	Hondo			Ryukyu		
				DAF	Normalized iHS	*P_iHS_*	DAF	Normalized iHS	*P_iHS_*
6	rs6907458	32138545	*MHC*	0.083	8.55	**1.20E**−**17**	0.018	5.36	8.20E−08
6	rs9268199	32278635		0.093	7.05	**1.78E**−**12**	0.190	9.66	**4.33E**−**22**
2	rs77756144	214137825	*IKZF2*	0.186	5.02	5.25E−07	0.314	5.60	**2.15E**−**08**

Bold: *P* < 6.33E−08.

CHR, chromosome; SNP, single-nucleotide polymorphism; BP, base pair position; DAF, derived allele frequency.

### Inspection of HLA Alleles Under Selection in the MHC region

Although statistical significance at the MHC region was observed in both Hondo and Ryukyu by iHS, the lead variants were different. Additionally, these variants showed substantial differences in allele frequencies (Table 2). To identify the HLA alleles potentially tagged by the lead variants, we carried out HLA imputation. HLA imputation has been demonstrated to accurately predict HLA alleles at a high resolution and has been commonly used as an alternative to traditional HLA typing for refining genetic association signals in the MHC region (Luo et al. 2021). This method has been instrumental in pinpointing HLA alleles or amino acid associated with numerous immune-related diseases (Raychaudhuri et al. 2012; Hu et al. 2015). This would also be the case to delineate selection signals. By this approach, we observed the lead HLA SNP in Hondo rs6907458 tags an extended haplotype: HLA-A*33:03-C*14:03-B*44:03-DRB1*13:02-DQB1*06:04-DPB1*04:01, and it has the strongest linkage disequilibrium (LD) with HLA-DQB1*06:04 (*r^2^* = 0.94), while in modest LD with the previously reported HLA-DPB1*04:01 (*r^2^* = 0.48) (Yasumizu et al. 2020) (Supplementary Table S4, Methods). In Ryukyu, the lead SNP rs9268199 tags DQB1*06:02-DRB1*15:01 (*r^2^* = 0.79). We noticed the HLA alleles under selection were among the alleles showing the biggest differences in frequencies between Hondo and Ryukyu (Supplementary Fig. S6 and Supplementary Table S5). Given that the *P_iHS_* is an approximate value and differences in *P_iHS_* of the lead variants can result from the normalization of iHS across different AF bins, we further examined both raw and normalized iHS for variants in the HLA region. Our analysis demonstrated rs6907458 and rs9268199 showed comparable scores, suggesting that these variants were likely subjected to selection in both subpopulations. Therefore, the differences in *P*-values should not be considered as evidence for differential selection (Supplementary Fig. S7).

### A Novel Signal at *IKZF2* Highlighted a Population-specific Variant

A genome-wide significant natural selection signal driven by rs77756144 (2q34) was detected in Ryukyu with a comparable approximate *P_iHS_* value in Hondo. The rs77756144 lies in-between the gene *IKZF2* and *SPAG16*, among which the *IKZF2* appears to be a probable candidate targeted for selection given its involvement in HTLV-1 infection related leukemia. *IKZF2* belongs to the Ikaros transcription factor family and played a critical role in lymphocyte development (Park et al. 2019) and the genetic region spanning *IKZF2* is frequently deleted in the HTLV-1 associated adult T cell leukemia/lymphoma (ATL). We observed an unusual AF pattern for rs77756144 in the gnomAD dataset (Karczewski et al. 2020), Northeast Asian Reference Database (NARD) (Yoo et al. 2019), and GenomeAsia 100K dataset (GenomeAsia100K Consortium et al. 2019). The allele was common in East Asian (AF ∼ 0.08) and Latino/admixed American populations (AF ∼ 0.135), but rare in European (AF = 0.001), African (AF = 0.002), and South Asian (AF = 0.01) (Supplementary Table S6). This suggests the derived allele may have originated in the Asian lineage before the split of East Asian and Native American. In our dataset, the AF is 0.186 in Hondo and strikingly reaches 0.31 in Ryukyu. We attempted to investigate if rs77756144 was a potential eQTL in the GTEx database. However, this variant was excluded from the analysis due to its low frequency among GTEx subjects (since most GTEx subjects had European ancestry).

### Validation of the iHS Signals by the Public-available 1KGP Data

We validated implicated iHS signals using the external 1000 Genomes Project (1KGP) WGS data. The extended haplotype homozygosity (EHH) analysis showed longer haplotype for rs6907458 (lead MHC variant in Hondo) in the Japanese compared with Chinese Han Beijing, the European and African populations (Supplementary Fig. S8). For *IKZF2* locus, we confirmed the lead variant rs77756144 was monomorphic in Europeans and African, while the derived haplotype extended longer in the Japanese compared with Chinese (Supplementary Fig. S9), which might indicate a higher level of selection pressure.

### Selection Profiles Revealed by FastSMC Analysis

**Fig. 3.**
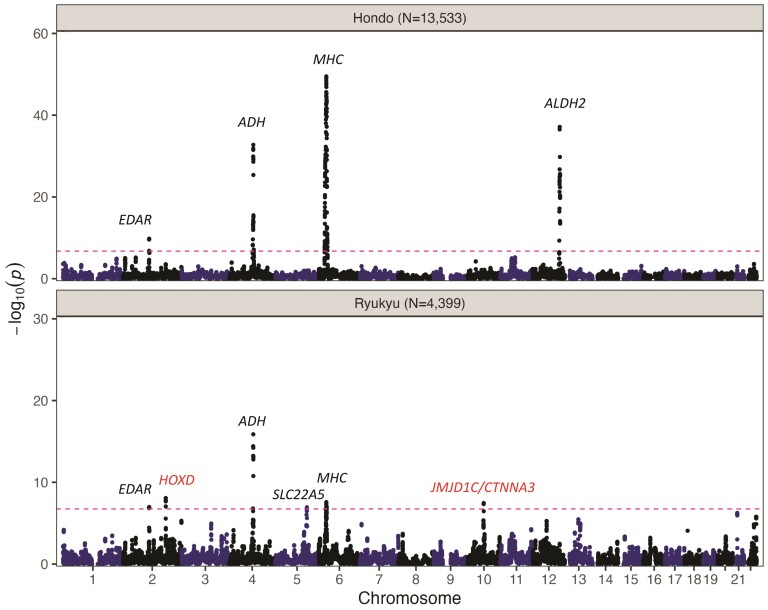
Candidate loci influenced by positive selection of the Japanese population in the past 150 generations based on FastSMC analysis in Hondo (top) and Ryukyu (bottom). The –log10(*P*_DRC150_) value (*y* axis) and the chromosomal position (*x* axis) of each binned region (0.05 cM) are plotted across the genome. The red dashed line represents the genome-wide significance threshold, which was obtained after Bonferroni correction for the number of bins of regions, subpopulations, and timescale (*P* < 1.80 × 10^−7^). Previously reported candidate genes linked with positive selection are indicated in black, while novel ones are highlighted in red.

To explore potential selection signals, we conducted FastSMC analysis at three different timescales within the past 150, 50, and 20 generations in Hondo and Ryukyu. Similarly, we conducted FastSMC for each Ryukyu subpopulation as a subanalysis. A timescale of 150 generations was chosen in order to make the results comparable with a previous study that employed the ASMC method (Yasumizu et al. 2020). We selected timescale of 50/20 generations to detect recent selection signatures. This is based on evidence that individuals from the Kofun era (250–538 AD) had similar genetic makeup to present-day Hondo Japanese (Cooke et al. 2021), indicating limited genetic changes in Hondo since then. The density plots and QQ plots depicting the empirical null model suggested the Gamma fitting in general is reasonable but might not handle large Density of Recent Coalescence (DRC) statistic values well, which would lead to conservative approximate *P*-values (Supplementary Fig. S10). After excluding loci that overlapped with known SVs or were flanked by segmental duplications, as these loci are prone to false-positive findings (see Methods and Supplementary Table S7), we identified 4 and 6 candidate loci, in Hondo and Ryukyu, respectively, that surpassed the genome-wide significance threshold based on the DRC_150_ statistic, which indicate these loci may have been influenced by selection within the past 150 generations (Table 3 and Fig. 3). Overall, we detected similar landscape of positive selection pressure between the Hondo and Ryukyu subpopulations. We observed signals from the MHC, the ADH cluster, and *EDAR* in both subpopulations, *ALHD2* in Hondo, and two novel loci: the HOXD cluster and *JMJD1C/CTNNA3*, specifically in Ryukyu. One difference was the presence of the *ALDH2* signal only in Hondo, which seemed to be consistent to the iHS results. Both *ALDH2* and *ADH* are key genes in alcohol metabolism and have been reported to be under positive selection in Japanese and Chinese populations (Okada et al. 2018; Yasumizu et al. 2020; Cong et al. 2022). To confirm the lack of a significant peak at *ALDH2* in Ryukyu was not due to a smaller sample size, we conducted the FastSMC to calculate the DRC_150_ statistic by down-sampling Hondo subjects to match the size of Ryukyu (*N* = 4,458), in which the *ALDH2* peak could be readily detected (Supplementary Fig. S11). Additionally, it is worth noting that all peaks in the initial analysis were detected with a smaller but comparable approximate *P*-value (Supplementary Table S8). This suggests that a smaller sample size may be sufficient for FastSMC analysis. We further inferred the selection signatures during the past 50 and 20 generations and observed differences in the selection profiles between Hondo and Ryukyu (Supplementary Figs. S12 and S13; Supplementary Table S9). There were differences in the selection signal on the MHC region between Hondo and Ryukyu over the past 50 and 20 generations. Additionally, two alcohol-related gene regions, *ALDH2* and *ADH*, demonstrated continued evidence of selection in Hondo. Furthermore, we noticed a signal from *SLC22A5* was present in Ryukyu but not in Hondo. The *SLC22A5* gene encodes a transporter that participates in the uptake and recycling of carnitine, and this gene had been previously reported to be involved in selection in Europeans (Rees et al. 2020). We conducted a subanalysis focusing on four Ryukyu subpopulations and found that the results were generally consistent with the overall findings in Ryukyu (Supplementary Figs. S14 to S18; Supplementary Table S10). Moreover, we observed an additional peak including *TAFA5*, 21q21.1, and *COLEC11* specific to certain subpopulations (Supplementary Figs. S16 and S18).

**Table 3:** Candidate loci detected to be under significant positive selection by FastSMC in Hondo and Ryukyu within the past 150 generations

CHR	Position (Mb)	Cytoband	*PDRC150*	Candidate Gene(s)	Group
2	108.623 to 108.927	2q12.3	1.61E−10	*EDAR*	Hondo
4	97.783 to 101.940	4q23	1.77E−33	ADH cluster	
6	24.401 to 36.091	6p21	3.40E−50	*MHC*	
12	110.271 to 113.954	12q24	7.25E−38	*ALDH2*	
2	108.676 to 108.927	2q12.3	1.05E−07	*EDAR*	Ryukyu
2	177.549 to 177.839	2q31.1	8.39E−09	HOXD gene cluster	
4	98.596 to 100.505	4q23	1.34E−16	ADH cluster	
5	130.414 to 131.774	5q23.3-q31.1	1.12E−07	*SLC22A5*	
6	31.058 to 32.658	6p21	2.59E−08	*MHC*	
10	66.619 to 67.091	10q21.3	3.39E−08	*JMJD1C, CTNNA3*	

Density of Recent Coalescence statistic within the past 150 generations.

CHR, chromosome; DRC150.

### Replication of FastSMC Signals in Independent Dataset

We conducted a replication study for significant signals observed in Hondo by FastSMC using an independent dataset of 12,103 Japanese individuals (genotyped on Illumina HumanOmniExpressExome BeadChip (OEE) microarray) (Supplementary Note S3). All significant loci detected in Hondo (*ADH*, *MHC*, *ALDH2*, and *EDAR*) were replicated with *P_DRC150_* < 2.32 × 10^−5^ (Supplementary Fig. S19). Finally, we confirmed that 28 out of 29 reported loci, based on the ASMC method in a previous study with independent samples from the BBJ dataset, showed a *P_DRC150_* < 0.05 in our replication dataset (Supplementary Table S11) (Yasumizu et al. 2020). The high replication rate demonstrates the robustness of the analysis and may indicate that the detection of novel candidate targets could be a result of including Ryukyu samples or East Asian-specific probes in the ASA.

## Discussion

Here we performed large-scale genome-wide scans to identify positive selection signals in the Japanese population using the DNA microarray designed to contain many EAS-specific variants. Precise genotype information of EAS-specific variants enabled us to show previously unseen genetic structures in the Japanese and uncovered four major island groups in the Ryukyu cluster. We identified one novel candidate locus at *IKZF2* by iHS and additional 8 candidate loci which might be influenced by positive selection within the past 20–150 generations. We found different selection signals in the MHC region and *ALDH2* region between the two subpopulations.

The iHS analysis identified significant variants in the MHC region that tags specific HLA alleles. In Hondo, the lead variant rs6907458 has the strongest LD with DQB1*06:04 and tags an extended haplotype HLA-A*33:03-C*14:03-B*44:03-DRB1*13:02-DQB1*06:04-DPB1*04:01, which spans from the MHC class I to class II region. This haplotype has also been previously implicated in positive selection within the Japanese population, with specific attention given to DPB1*04:01 (Kawashima et al. 2012; Yasumizu et al. 2020). It is noteworthy that the HLA-A*33:03-C*14:03-B*44:03-DRB1*13:02-DQB1*06:04-DPB1*04:01 haplotype was reported to be the second most frequent haplotype in the mainland Japanese, and the most frequent haplotype in Korean, but rare or not observable in other East Asian populations (Nakaoka and Inoue 2015; Zhou et al. 2015; Park et al. 2016). Given its population specificity, the long haplotype range and the constituent alleles are in strong LD, it has been speculated that this haplotype originated in Korean Peninsula, and then likely spread to Japan Hondo, potentially during the Yayoi period, followed by a rapid expansion (Nakaoka and Inoue 2015). This speculation seems to be compatible with the inference based on the FastSMC method, which revealed strong signals in the MHC region in Hondo especially within the past 20–50 generations. We reported a MHC signal in Ryukyu, in which the lead positively selected MHC variant rs9268199 tags two HLA alleles: HLA-DRB1*15:01 and HLA-DQB1*06:02, which are in tight LD within the MHC class II region. The *DRB1:15:01* exhibited the highest frequency among Oceania, southeast Asia based to the survey of HLA frequency worldwide (http://www.allelefrequencies.net/) (Isshiki and Ohashi 2020).

Through a literature review, we found some examples that HLA alleles potentially under selection pressure are associated with various medical conditions, as reported in genetic association studies (Nishida et al. 2016; Pugliese et al. 2016; Yasunami et al. 2017; Penova et al. 2021). Some of these alleles could potentially offer protection against specific viral infections or conditions related to viral infections (Nishida et al. 2016; Penova et al. 2021). This suggests that viruses may be one of the contributing factors for the observed selection signals. The HLA allele DQB1*06:04, which is most strongly tagged by lead variant rs6907458 in Hondo, had been reported with protective effects with Hepatitis B virus (HBV) infection in a previous study (reported *P* = 2.73 × 10^−5^, OR = 0.44) (Nishida et al. 2016). The estimated prevalence of chronic HBV infection in Japan in 2016 was around 0.6% (Razavi-Shearer et al. 2018), while historically, the prevalence of chronic HBV infection was much higher before the HBV vaccination program was implemented nationwide in 1986. As such, there may be a potential connection between the positive selection of DQB1*06:04 and HBV infection. For DRB1*15:01-DQB1*06:02, it has been recognized as the most potent genetic factor for narcolepsy (Miyagawa and Tokunaga 2019). Recent research has also reported an association of this haplotype with an increased risk of systemic lupus erythematosus (SLE) and a protective effect against type 1 diabetes (T1D) (Pugliese et al. 2016; Kawasaki et al. 2023). In addition, a GWAS of HTLV-1-associated myelopathy/tropical spastic paraparesis (HAM/TSP) patients and asymptomatic HTLV-1 carriers identified HLA-DRB1*15:01 (reported *P* = 1.06 × 10^−5^, OR = 0.59) and HLA-DQB1*06:02 (reported *P* = 1.78 × 10^−6^, OR = 0.43) as top protective alleles (Penova et al. 2021). This raises the possibility of a potential relationship between the HTLV-1 and the selection signal. The HTLV-1 infection is prevalent in Japan (Watanabe 2011). In particular, Ryukyu and the southwestern part of Hondo have the highest HTLV-1 infection rate (Iwanaga 2020). The HTLV-1 infection is largely latent but can reactivate and lead to severe symptoms such as HAM/TSP which lead to neuronal damage or even life-threatening ATL. The HLA-DRB1*15:01-DQB1*06:02 haplotype has been reported to be under positive selection in Papua New Guinea, a region known for its high prevalence of HTLV-1 (Gessain and Cassar 2012). Additionally, it is noteworthy that Oceania and Southeast Asia, where the haplotype exhibits a relatively higher frequency, are recognized for their elevated HTLV-1 prevalence (Gessain and Cassar 2012). The haplotype frequency of DRB1*15:01 and DQB1*06:02 is significantly higher in Ryukyu compared with that of Hondo (0.191 vs. 0.071, *P* = 1.33 × 10^−179^). While this may mirror the differences in HTLV-1 prevalence between Ryukyu and Hondo, we should not overinterpret the potential correlation between HLA allele frequency and HTLV-1 prevalence. Factors other than these pathogens, diseases or demographic history could also contribute to the difference in HLA allele frequencies and further accumulation of investigations is essential. Moreover, the implicated HLA alleles may have associations with other pathogens. For example, DRB1*15:01 has been reported as a co-receptor for the Epstein–Barr virus (Menegatti et al. 2021), which implies that this allele could potentially interact with other known or unidentified viruses.

In addition to MHC, the iHS analysis identified another strong signal at *IKZF2* which might be linked with the HTLV-1. The IKZF2 has been recently shown as a key regulator of T cell development, which maintains the stem cell self-renewal and suppresses myeloid linage differentiation by modulating chromatin accessibility (Park et al. 2019). In a multiomics study consisted of 426 ATL cases, intragenic deletions and inversions of *IKZF2* were observed as one of the most frequent genetic alterations in ATL (Kataoka et al. 2015), suggesting it might play a critical role in pathogenesis of ATL. It is still unknown whether rs77756144 has any protective effects against HTLV-1 infection outcomes, and further research is needed to uncover the biological significance of this candidate selection signal.

Based on a recently developed method FastSMC, we detected additional candidate targets that may have been influenced by selection in the Japanese population at three different time frames. The identified loci include *EDAR*, *ADH* cluster, and *ALDH2*, three well-known East Asian-specific loci targeted by positive selection (Fujimoto et al. 2008; Kimura et al. 2009; Okada et al. 2018; Yasumizu et al. 2020). These *ADH* and *ALDH2* are related to alcohol metabolism and missense variants rs1229984 (Arg48His) in *ADH1B* and rs671 (Glu504Lys) in *ALDH2*, which make carriers less tolerant to alcohol, were shown to be favored by selection (Yasumizu et al. 2020). The FastSMC analysis, focusing on the past 20–150 generations, detected a signal at *ADH* in both Hondo and Ryukyu, whereas a signal for *ALDH2* was observed only in Hondo and not in Ryukyu. This difference warrants further investigation in future analyses. The seemingly different selection profiles for *ADH* and *ALDH2* may be consistent with a recent study, which suggests that the onset of positive selection for *ADH* occurred approximately 12,500 yr earlier than that for *ALDH2*. Specifically, while positive selection on *ADH* may have begun around 20,000 yr ago, that selection on *ALDH2* is estimated to have started about 7,500 yr ago in East Asian populations (Kawai et al. 2023). The reasons for the positive selection of these alcohol-related genes remain unknown, but it has been proposed that they may be related to the large-scale adoption of rice cultivation in East Asia (Supplementary Note S4).

We highlight several aspects where caution should be exercised. First, genome-wide significant signals from MHC based on DRC_50/20_ were observed in Hondo but not in Ryukyu. This result requires careful interpretation; it does not necessarily indicate a lack of natural selection in the MHC in Ryukyu within the past 50 and 20 generations, a conclusion that would contradict the iHS results. Instead, the HLA alleles under selection in Hondo may originate differently given the peopling history of Hondo as we previously discussed. Second, it should be emphasized that difference in the strength of signature should not be interpreted as differential selection. The detection of a selection signature depends on multiple factors such as allele frequency and the population's demographic history, and potential technical artifact (Meyer et al. 2006; Nielsen et al. 2007; Huber et al. 2014). Thus, lack of significance or diminished evidence in a subpopulation should not be taken as sufficient proof of differential selection. Third, we recommend conducting replication analyses of the newly identified candidate loci in future studies, utilizing independent datasets.

In summary, we presented genome-wide scans of selection in the Japanese population with individuals collected from both Hondo and Ryukyu. We highlighted selection signatures in the MHC region. The selection signal in Ryukyu might be linked to specific diseases or viral infections such as HTLV-1, although further analysis will be required to validate this hypothesis. The FastSMC analysis detected signals for the *ADH1B* and *ALDH2* genes for the Hondo and Ryukyu populations, and nominated novel candidate selection targets for future studies. Our study highlights the significance and need for broadening previous genetic studies to include individuals from a range of genetic backgrounds. Furthermore, it underscores the value of considering population-specific variants and connecting selection signals with epidemiological characteristics.

## Methods

### Samples and Genotype Data

Samples analyzed in this study were obtained from two cohorts. The first cohort consisted of 13,753 participants who were recruited from the NCGG Biobank of Japan. The second one consisted of 6,613 participants who were recruited at Okinawa Prefecture through the OBi Project. All genomic DNA samples were genotyped by Illumina Infinium ASA v1.0. Origins of individual participants were surveyed in OBi Project and information of the birthplace (islands) for their four grandparents were obtained by questionnaire. The geographic birthplace information was not available for the NCGG cohort. The genotyping was performed following the manufacturer's recommended protocols. After the merge of raw genotyping calls of the two cohorts, we conducted QC to remove variants that are low quality and samples with low call-rates, having first or second degree relative(s), or non-Japanese inferred by PCA.

### PCA and PCA-UMAP

The PCA was conducted to explore and visualize underlying population structure. To make appropriate inferences, we included additional samples from two public datasets: (1) 2,504 samples in the 1KGP (1000 Genomes Project Consortium 2012) and (2) 85 Koreans from the Korean Personal Genome Project (KPGP) (http://kpgp.kr/). For the former, we obtained the high-depth WGS released by the New York Genome Center (NYGC) (https://www.internationalgenome.org/data-portal/data-collection/30x-grch38), and extracted SNPs included in the ASA and manually lift-down to hg19 based on the manifest files provided by Illumina (https://jp.support.illumina.com/downloads/infinium-asian-screening-array-v1-0-product-files.html). The genotype data of Korean was downloaded from the following site: http://camda2021.bioinf.jku.at/kpg_prepro. After the merge of all samples, we pruned the non-HLA variants and selected pruned common variants (MAF >= 5%; pruning parameter: index-pair 100, 10, 0.2), and the top 20 PC scores were calculated by PLINK v1.9 (Chang et al. 2015). To gain a higher resolution of the population structure, we further applied UMAP) analysis for the top 20 PCs, using the R package umap (0.2.7.0) (McInnes et al. 2018).

### *F_ST_* Calculation

We calculated the Hudson's *F_ST_* value of each SNP using the KRIS software package. (version 1.1.1, R version 3.6.2; https://rdrr.io/cran/KRIS/). We used modified function “fst.hudson” for calculation of *F_ST_* with standard error. Since the distribution of weighted mean *F_ST_* theoretically follows the Chi-square distribution (Hartl and Clark 1997), we fitted these two distributions and calculated the approximate one-sided *P*-values. To correct the multiple testing, we adopted Bonferroni adjusted genome-wide significance level at *P* = 1.20 × 10^−7^ (0.05/415,141 SNPs).

### Integrated Haplotype Score Analysis

We restricted the analysis to variants whose ancestral allele is supported by chimp and macaque. Ancestral states of each SNP were inferred using the ancestral hg19 genome provided by the 1000 genomes consortium, based on the human–chimp–macaque alignment (http://ftp.1000genomes.ebi.ac.uk/vol1/ftp/phase1/analysis_results/supporting/ancestral_alignments/) (Flicek et al. 2012). To ensure accurate phasing and iHS analysis, we utilized the Japanese-specific recombination map, which was created using the 1KGP JPT WGS data (Spence and Song 2019) (https://github.com/popgenmethods/pyrho#human-recombination-maps). Since the Ryukyu population was not present in the 1KGP dataset, we used the same recombination map of JPT. The SNP phasing was done for each chromosome using Eagle v2.4.1 with the default parameters (Loh et al. 2016). We excluded variants which have a MAF < 1% or whose ancestral alleles were not determined. We interpolated the physical position for each variant. We then conducted iHS analysis using the software selscan (v1.3) (Szpiech and Hernandez 2014) for the Hondo and Ryukyu clusters inferred by PCA-UMAP. The normalized iHS (standardized *Z* scores) were obtained by normalization under 100 MAF bins. Approximate *P-*values were calculated by fitting the normalized iHS scores, assuming a normal distribution. To correct the multiple testing, we adopted Bonferroni adjusted genome-wide significance level at approximate *P_iHS_* = 6.33 × 10^−8^ (0.05/394,906/2, which is the number of variants that received an iHS score and two subpopulations) in the main analysis, and approximate *P_iHS_* = 3.17 × 10^−8^ (0.05/394,906/4) in the subanalysis for each Ryukyu cluster. EHH around the selected SNPs were calculated and plotted using the rehh software package (Gautier and Vitalis 2012) (version 3.2.2, R version 3.6.2) with the public available 1KGP data (http://ftp.1000genomes.ebi.ac.uk/vol1/ftp/release/20130502).

### HLA Imputation

To understand variations in HLA alleles depending on regional populations defined in PCA-UMAP, we conducted HLA imputation with the HLA-TAPAS pipeline (Luo et al. 2021). In brief, we extracted 7,885 SNPs of the HLA region (chr6:25MB-34MB, hg19). Based on a reference panel build from 1KGP dataset, the HLA alleles were imputed up to four digits using an enhanced version of SNP2HLA (Jia et al. 2013). We then calculated the frequencies of HLA alleles in each population. We also calculated the LD *r*-squared (*r2*) between the top SNPs under selection and HLA alleles using PLINK v1.9.

### FastSMC Analysis

In addition to iHS, we attempted to use FastSMC (Nait Saada et al. 2020), an algorithm designed for Identical-By-Descent (IBD) detection and estimation of coalescent times for each IBD, to specifically identify recent selection signals in the Japanese population. By analyzing locus-specific IBD sharing patterns, we calculated DRC within past 20, 50, and 150 generations (DRC_20_, DRC_50_, and DRC_150_) from IBD quality scores. We summarized the mean value for each sliding window at a size of 0.05 centimorgan (cM). The decoding file was prepared from the JPT demographic and allele frequency file. The higher DRC values are expected to be found in the genomic loci that were shared by many subjects but inherited from limited number of common ancestors, which might be due to the recent positive selection. We fitted a Gamma distribution to the estimated DRC values using the neutral regions in the genome. We first excluded genetic loci that have been reported in the literature to be under positive selection in the Japanese population, and we iteratively removed regions that showed evidence of being targeted by selection based on the DRC statistic (Fujimoto et al. 2008; Okada et al. 2018; Yasumizu et al. 2020). Based on this null model, we derived approximate one-sided *P*-values. To correct the multiple testing, we adopted Bonferroni adjusted genome-wide significance level at approximate *P_DRC_* = 1.80 × 10^−7^ (0.05/46,306 regions/2 subpopulations/3 time points) in the main analysis, and at approximate *P_DRC_* = 9.00 × 10^−8^ (0.05/46,306 regions/4 Ryukyu subpopulations/3 time points) in the subanalysis.

### Inspecting and Filtering Potential False-positive Significant Loci

Extended LD and shared haplotypes can also result from the underlying presence of SV, which may suppress recombination in heterozygote carriers (Fishman et al. 2013; Morgan et al. 2017). To examine whether any loci deemed to have genome-wide significance by iHS or DRC statistic overlap with known SVs, we examined the Human Genome Structural Variation Consortium (HGSVC) Phase 2 dataset (Ebert et al. 2021). We obtained the called SV data of 3,202 deep-coverage samples processed by the PanGenie genotyping pipeline (http://ftp.1000genomes.ebi.ac.uk/vol1/ftp/data_collections/HGSVC2/release/v1.0/PanGenie_results/pangenie_merged_multi_nosnvs.vcf.gz). Given that the genome build of the SV data is in hg38, we liftovered the genomic positions for all significant loci using liftover tool from the UCSC genome browser (https://genome.ucsc.edu/cgi-bin/hgLiftOver). We extracted common SVs with length >=10 kb and an AF >= 1% and in Asians. To detect any significant loci overlapping with the SVs, we employed the intersectBed tool from Bedtools (v2.3). Specifically, we considered loci that had an overlap of at least 10% of the length of the SV. Utilizing the “Segmental Dups” track and “Reference Assembly Fix Patch Sequence Alignments” track, both available via the UCSC genome browser (https://genome.ucsc.edu), we identified and subsequently excluded significant loci that were either encompassed by or flanked by segmental duplications or contained an excessive number of reference “fix patches’.

### Replication Study

We additionally included 12,103 Japanese samples from the BBJ that were genotyped by Illumina OmniExpressExome assay. These samples have not been previously analyzed and were independent of 170,882 BBJ samples used in the previous genome-wide scan for selection signals in the Japanese population (Yasumizu et al. 2020). This dataset was pre QCed and used in a previous study (Ito et al. 2023). We obtained the phased VCF files (Supplementary Note S3). The FastSMC analysis was conducted in the same manner as the previously described for ASA data. The stringent Bonferroni adjusted genome-wide significance levels were set at *P* = 1.13 × 10^−6^ (0.05/44,294 regions).

## Supplementary Material

Supplementary material is available at *Molecular Biology and Evolution* online.

## Supplementary Material

msad231_Supplementary_Data

## Data Availability

The summary statistics of the iHS and FastSMC analysis is available from the Japanese ENcyclopedia of GEnetic associations by RIKEN (JENGER) website (http://jenger.riken.jp/).
