# Supplementary material for: Natural Selection Signatures in the Hondo and Ryukyu Japanese Subpopulations
Source: Mol Biol Evol. 2023 Oct 30;40(10):msad231. doi: 10.1093/molbev/msad231 (PMC10615566; doi:10.1093/molbev/msad231)
Supplement: msad231_Supplementary_Data [file msad231_supplementary_data.zip › MBE-23-0248.R2.SupNote.final.pdf]

# Supplementary Materials for

## Natural selection signatures in the Hondo and Ryukyu Japanese sub-populations

Xiaoxi Liu, Masatoshi Matsunami, Momoko Horikoshi, Shuji Ito,

Yuki Ishikawa, Kunihiko Suzuki, Yukihide Momozawa, Shumpei Niida, Ryosuke Kimura,

Kouichi Ozaki, Shiro Maeda, Minako Imamura*, Chikashi Terao*

*Corresponding author

Email: mimamura@med.u-ryukyu.ac.jp, chikashi.terao@riken.jp

**This PDF file includes:**

Supplementary Notes 1 to 4

Supplementary Reference

Supplementary Figures 1 to 19

**Other Supplementary Material for this manuscript includes the following:**

Supplementary Tables 1 to 11 (separate Excel file)

# Supplementary Notes

## Supplementary Note 1: The geography of Japan and the peopling history

Japan is situated in the Far East, off the coast of the Eurasian Continent. The country is composed of several thousand islands, including the Japanese Archipelago and the Ryukyu Archipelago. In a narrow definition, the Japanese Archipelago includes four main islands: Honshu, Shikoku, Kyushu, and Hokkaido, and excludes the Ryukyu Archipelago. These four main islands are collectively referred to as Hondo. During the Pleistocene epoch, Hondo was connected to the Asian continent via a land bridge on three separate occasions: approximately 1 million years ago (Ma), 500 thousand years ago (ka), and 300 ka. Additionally, the northern part of Hokkaido was connected to the continent via Sakhalin Island during the last glacial period, around 100-10 ka, and has been isolated since (Taira et al. 2016). Likely due to this geographic isolation, human settlement in Japan occurred relatively later (around 35 ka) compared to other regions of the Asian continent. The first inhabitants were the Jomon people, known for their unique pottery culture, which existed from 14,500 B.C. to 1000 B.C. Subsequently, groups commonly referred to as the Yayoi people migrated from the Asian continent and introduced rice farming between 1000 B.C. and A.D. 250 (Osada and Kawai 2021). Recent studies suggest that multiple waves of immigration occurred in Japan's pre-history (T. Jinam et al. 2021a; T.A. Jinam et al. 2021b). It has been proposed the dual-cluster genetic structure of present-day Japanese primarily reflects varying degrees of Jomon and Yayoi ancestries (Watanabe et al. 2020).

## Supplementary Note 2: The geography and the peopling of Ryukyu Archipelago

The Ryukyu Archipelago is the southernmost island of Japan, and mainly consists of the Amami, Okinawa, Miyako, and Yaeyama Islands. In addition to these islands, Kume-jima and Kerama Islands are also included in the Ryukyu Archipelago. Among these island groups, Okinawa, Miyako, Yaeyama, and Kume-jima/Kerama Islands are part of Okinawa Prefecture. About 1.45 million people are living in Okinawa prefecture. This archipelago is surrounded by the Pacific Ocean and the East China Sea. Therefore, the people on these islands are considered to have been isolated from other populations. The peopling in Ryukyu Archipelago has a unique history, compared with that in Japanese Archipelago. On the Ryukyu Archipelago, human bones dating to the Paleolithic period were excavated from several sites (Koganebuchi and Kimura 2019). It has been suggested that the Paleolithic people in the Ryukyu Archipelago are not the main ancestors

of modern Ryukyu people, but rather that modern Ryukyu people are descendants of migrations from the Japanese Archipelago in the Neolithic Period or later. In the Neolithic period when the Jomon culture reached the Okinawa Islands, however, there is a long archaeological blank in the record on the Miyako and Yaeyama Islands. After these blanks, the Shimotabaru culture (About 4.2-3.5 ka) and the Aceramic culture (2.5–0.9 ka) emerged and disappeared mainly in the Yaeyama and the Miyako Islands. These cultures are recognized as being distinct from Jomon culture on the Japanese mainland. These two different cultures were unified during the Gusuku period (0.9−0.5 ka). Especially, a recent study suggested that Miyako people were genetically divided into three populations, and these populations are derived from two migration waves that occurred at the Gusuku and more recent periods (Yasumizu et al. 2020).

**Supplementary Note 3: QC of the dataset for the replication study**

We extracted 12,103 Japanese samples from a pre-QCed dataset from the Biobank Japan (BBJ). The samples were genotyped on the Illumina HumanOmniExpressExome BeadChip. For sample QC, samples were excluded if they met the following criteria: (1) a sample call rate of <0.98; and (2) outliers from East Asian clusters identified by principal component analysis. For SNP QC, variants were removed if they meet any of the following criteria: (1) call rate < 99%; (2) HWE-P $< 1\times10^{-6}$. The phasing was done using SHAPEIT v4.2 (Delaneau et al. 2019).

**Supplementary Note 4: Hypotheses to explain the selective pressure on alcohol-related genes in East Asian**

The reasons for the positive selection of alcohol-related genes remain unclear. However, it has been proposed that this could be related to the large-scale adoption of rice cultivation in East Asia. One hypothesis suggests that elevated levels of acetaldehyde may confer a protective effect against parasitic infections, particularly from anaerobic and microaerophilic gut organisms like *Entamoeba histolytica* (*E. histolytica*) (Goldman and Enoch 1990; Oota et al. 2004). *E. histolytica* relies on a key bifunctional alcohol/aldehyde dehydrogenase protein, EhADH2, for its growth and survival, where this protein plays a vital role in the utilization of glucose as an energy source via the fermentative pathway (Espinosa et al. 2001; Strauss 2010). Elevated acetaldehyde levels in the blood due to *ALDH2* deficiency could potentially impair the function of EhADH2, offering a form of protection against infection. This implied notion could find

indirect support from the fact that nitroimidazole, an ALDH inhibitor, has been used as the first-line medication to treat E. histolytica infection (Azam et al. 2015). Should this hypothesis be validated, individuals carrying functional variants of *ALDH2* and *ADH* could benefit from potential resistance to parasites. If such an advantage exists, it may be especially relevant in East Asia, where farming has a long history of widespread practice. In this context, the likelihood of encountering parasites like *E. histolytica* may be elevated due to activities such as working in rice paddy, where anaerobic conditions in the soil can create an environment conducive to certain parasites. Additionally, the use of human or livestock excreta as fertilizer in agriculture can further increase the risk of exposure to pathogens (Tran-Thi et al. 2017). While other hypotheses exist, such as protection against hepatitis B virus (Zhang et al. 2021). Furthermore, survival analysis conducted on the BBJ dataset revealed that carrying functional variants of *ADH1B* and *ALDH2* had a positive impact on all-cause mortality in the Japanese population. Notably, these effects were independent of alcohol consumption (Sakaue et al. 2020).

## Supplementary Reference:


Azam A, Peerzada MN, Ahmad K. 2015. Parasitic diarrheal disease: drug development and targets. *Front. Microbiol.*

Delaneau O, Zagury J-F, Robinson MR, Marchini JL, Dermitzakis ET. 2019. Accurate, scalable and integrative haplotype estimation. *Nat Commun* 10:5436.

Espinosa A, Yan L, Zhang Z, Foster L, Clark D, Li E, Stanley SL. 2001. The Bifunctional Entamoeba histolytica Alcohol Dehydrogenase 2 (EhADH2) Protein Is Necessary for Amebic Growth and Survival and Requires an Intact C-terminal Domain for Both Alcohol Dehydrogenase and Acetaldehyde Dehydrogenase Activity. *Journal of Biological Chemistry* 276:20136–20143.

Goldman D, Enoch M-A. 1990. Genetic Epidemiology of Ethanol Metabolic Enzymes: A Role for Selection. In: Simopoulos AP, Childs B, editors. World Review of Nutrition and Dietetics. Vol. 63. S. Karger AG. p. 143–160.

Jinam T, Kawai Y, Kamatani Y, Sonoda S, Makisumi K, Sameshima H, Tokunaga K, Saitou N. 2021a. Genome-wide SNP data of Izumo and Makurazaki populations support inner-dual structure model for origin of Yamato people. *J Hum Genet.*

Jinam TA, Kawai Y, Saitou N. 2021b. Modern human DNA analyses with special reference to the inner dual-structure model of Yaponesian. *AS* 129:3–11.

Koganebuchi K, Kimura R. 2019. Biomedical and genetic characteristics of the Ryukyuans: demographic history, diseases and physical and physiological traits. *Annals of Human Biology* 46:354–366.

Oota H, Pakstis AJ, Bonne-Tamir B, Goldman D, Grigorenko E, Kajuna SLB, Karoma NJ, Kungulilo S, Lu R-B, Odunsi K, et al. 2004. The evolution and population genetics of the ALDH2 locus: random genetic drift, selection, and low levels of recombination. *Ann Human Genet* 68:93–109.

Osada N, Kawai Y. 2021. Exploring models of human migration to the Japanese archipelago using genome-wide genetic data. *Anthropological Science* 129:45–58.

Sakaue S, Akiyama M, Hirata M, Matsuda K, Murakami Y, Kubo M, Kamatani Y, Okada Y. 2020. Functional variants in ADH1B and ALDH2 are non-additively associated with all-cause mortality in Japanese population. *Eur J Hum Genet* 28:378–382.

Strauss E. 2010. Coenzyme A Biosynthesis and Enzymology. In: Comprehensive Natural Products II. Elsevier. p. 351–410. Available from: https://linkinghub.elsevier.com/retrieve/pii/B9780080453828001416

Taira A, Ohara Y, Wallis SR, Ishiwatari A, Iryu Y. 2016. Geological evolution of Japan: an overview. In: Moreno T, Wallis S, Kojima T, Gibbons W, editors. The Geology of Japan. Geological Society London. p. 1–24.

Tran-Thi N, Lowe RJ, Schurer JM, Vu-Van T, MacDonald LE, Pham-Duc P. 2017. Turning poop into profit: Cost-effectiveness and soil transmitted helminth infection risk associated with human excreta reuse in Vietnam.Booth M, editor. *PLoS Negl Trop Dis* 11:e0006088.

Watanabe Y, Isshiki M, Ohashi J. 2020. Prefecture-level population structure of the Japanese based on SNP genotypes of 11,069 individuals. *J Hum Genet*.

Yasumizu Y, Sakaue S, Konuma T, Suzuki K, Matsuda K, Murakami Y, Kubo M, Palamara PF, Kamatani Y, Okada Y. 2020. Genome-Wide Natural Selection Signatures Are Linked to Genetic Risk of Modern Phenotypes in the Japanese Population. *Molecular Biology and Evolution* 37:1306–1316.

Zhang X, Sun A, Ge J. 2021. Origin and Spread of the ALDH2 Glu504Lys Allele. *Phenomics* 1:222–228.

# Supplementary Figures

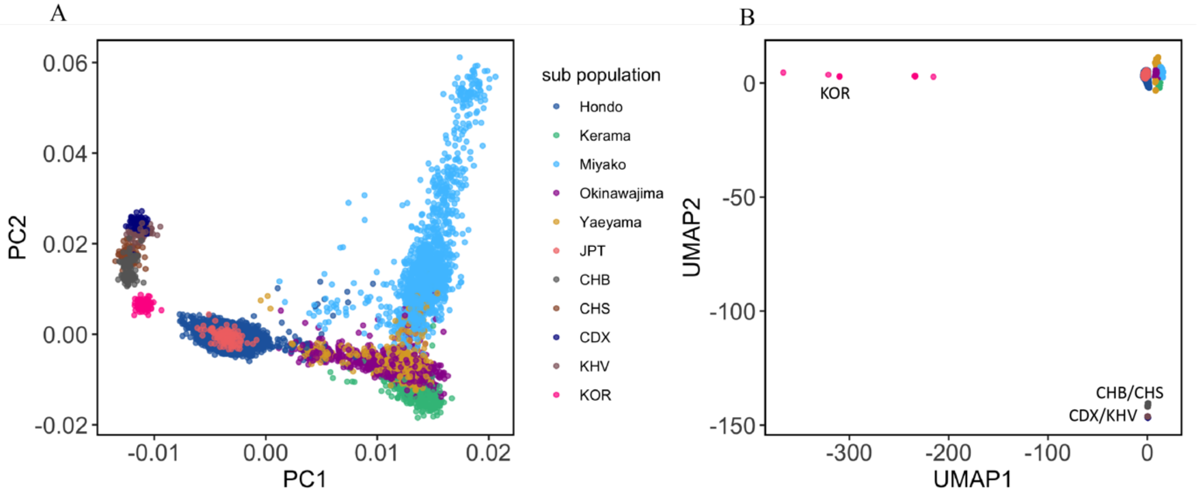


**Supplementary Figure 1 | PCA and PCA-UMAP plots with other East Asian populations. (A**) The PCA plot based on pruned common variants (MAF >= 1%) for the Japanese study samples (N=17,932) and other East Asian populations including north Han Chinese (CHB), south Han Chinese (CHS), Chinese Dai in Xishuangbanna (CDX) and Kinh in Ho Chi Minh City of Vietnam (KHV) derived from 1000 Genomes Project (1KGP) and the Korean (KOR) from the KPGP project. (**B)** The PCA-UMAP plot of the same samples as plotted in Fig. S1A

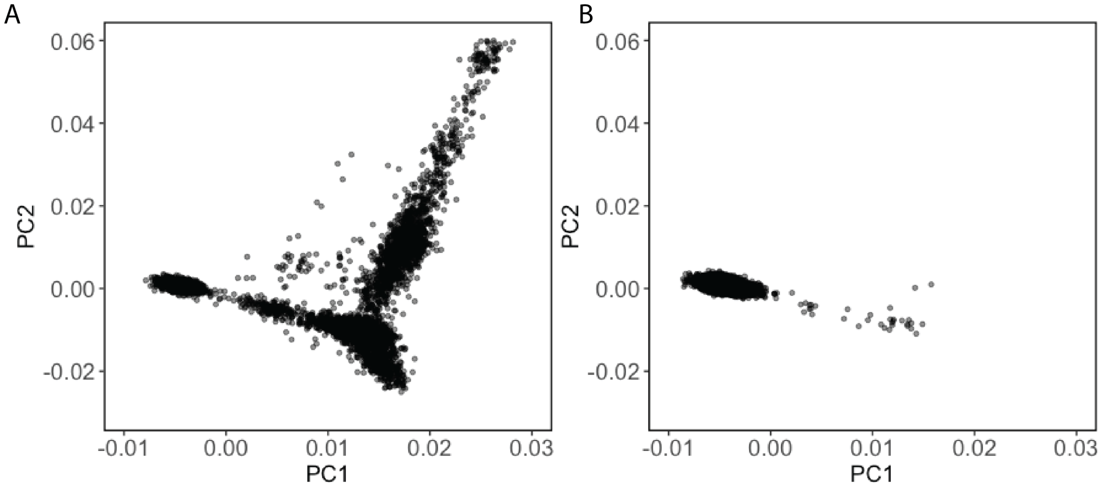


**Supplementary Figure 2 | Confirmation of the population structure not affected by any potential technical batch effect(s).**
(**A**) The PCA plot based on pruned common variants (MAF >= 1%) with samples in the OBi cohort (N=5,303) (**B)** The projection of PCA in (A) to NCGG cohort (N = 12,629). The NCGG samples were located into the expected position of Hondo cluster, implying the PCA shown in Fig 1C reflected the true population structures rather than due to any technical batch effects.

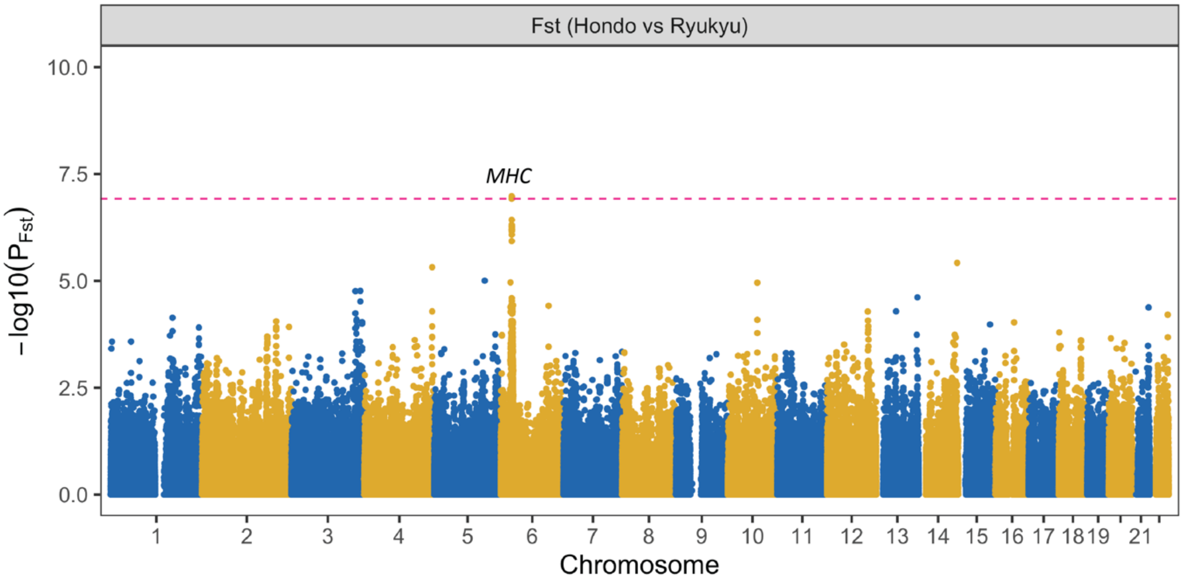


**Supplementary Figure 3 | Manhattan plot of the $P_{Fst}$ values (Hondo vs Ryukyu)**
The -log10($P_{Fst}$) value (*y*-axis) and the chromosomal position (*x*-axis) of each SNP is plotted across the genome. The red dashed line represents the genome-wide significance threshold, which was obtained after Bonferroni correction for the number of variants tested ($P_{Fst} < 1.20\times10^{-7}$).

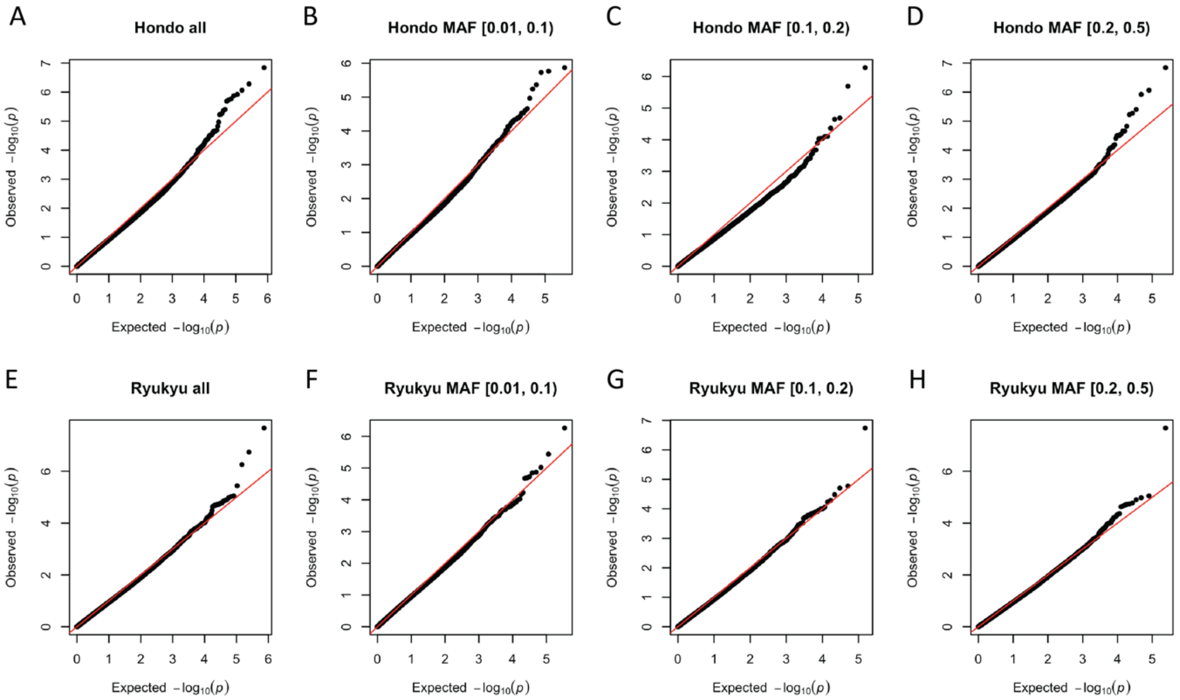


**Supplementary Figure 4 | Quantile-quantile (QQ) plots of the iHS statistics in non-HLA regions**

Comparison of observed approximate $P_{iHS}$ values versus expected $P$ values for all variants in non-HLA regions, stratified by minor allele frequency (MAF) bins, in Hondo (panels **A-D**) and Ryukyu (panels **E-H**).

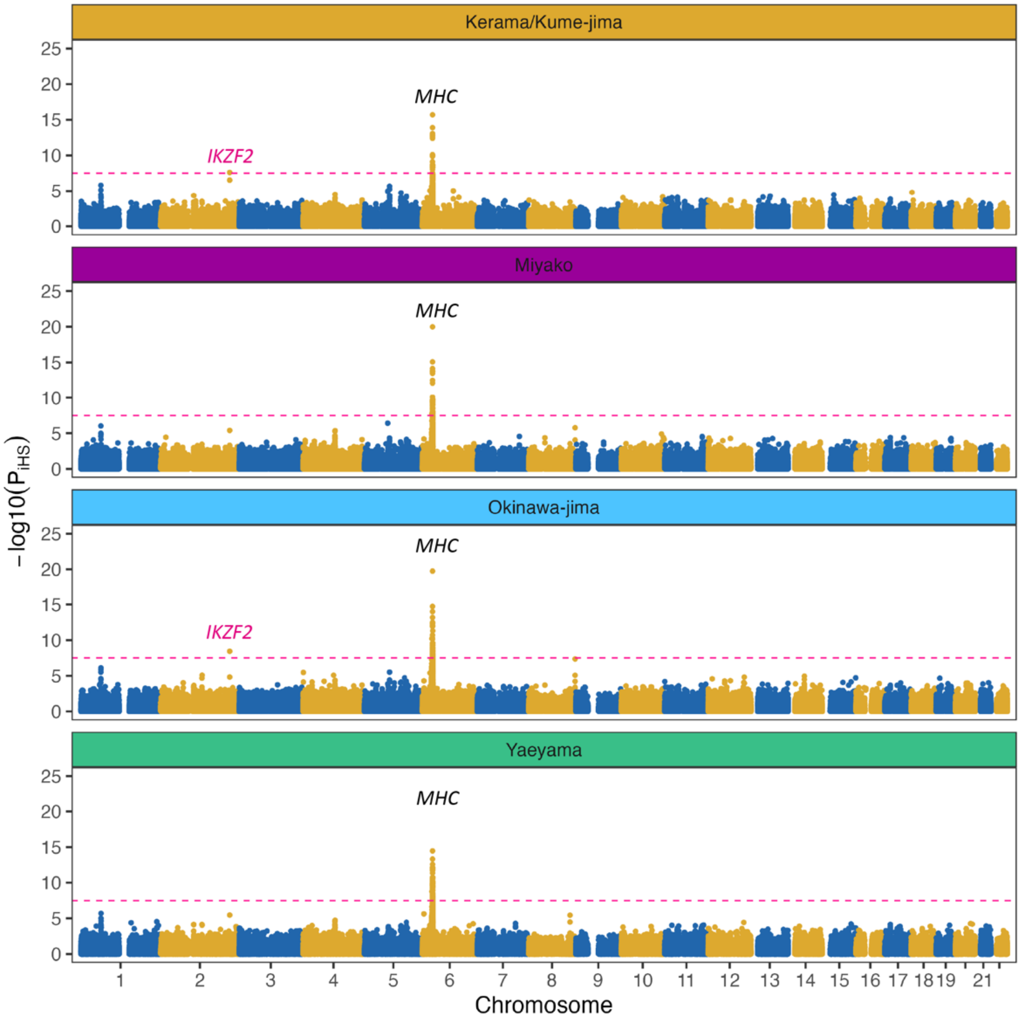


**Supplementary Figure 5 | Manhattan plots of the positive selection signals based on iHS analysis in four Ryukyu Island groups: Kerama/Kume-jima, Miyako, Okinawa-jima and Yaeyama.** The -log10($P_{iHS}$) value (y-axis) and the chromosomal position (*x*-axis) of each SNP are plotted across the genome. The red dashed line indicates the Bonferroni-corrected genome-wide significance threshold (approximate $P_{iHS} < 3.17 \times 10^{-8}$).

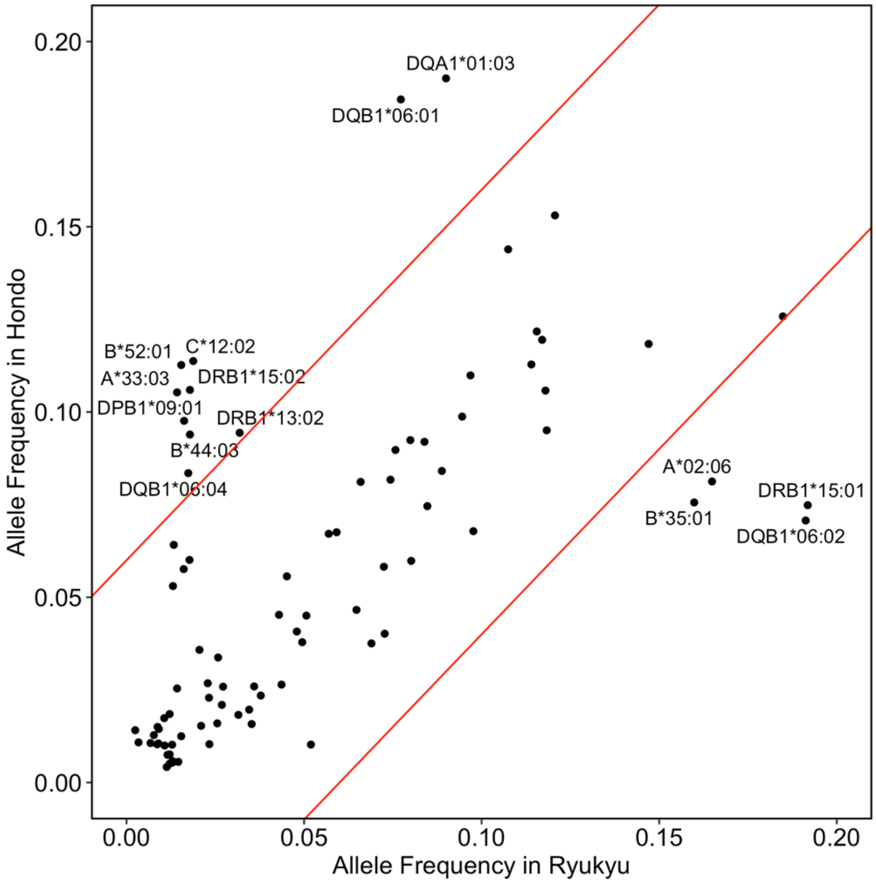


**Supplementary Figure 6 | The Allele frequencies of HLA alleles in Hondo and Ryukyu.** HLA alleles with AF > 1% and AF <= 20% were plotted (N=89). The x-axis represents the AF in the Ryukyu population, while the y-axis represents the AF in the Hondo population. Alleles that exhibited an AF difference of at least 5% between Ryukyu and Hondo were annotated. The red lines on the plot indicate the 5% difference threshold.

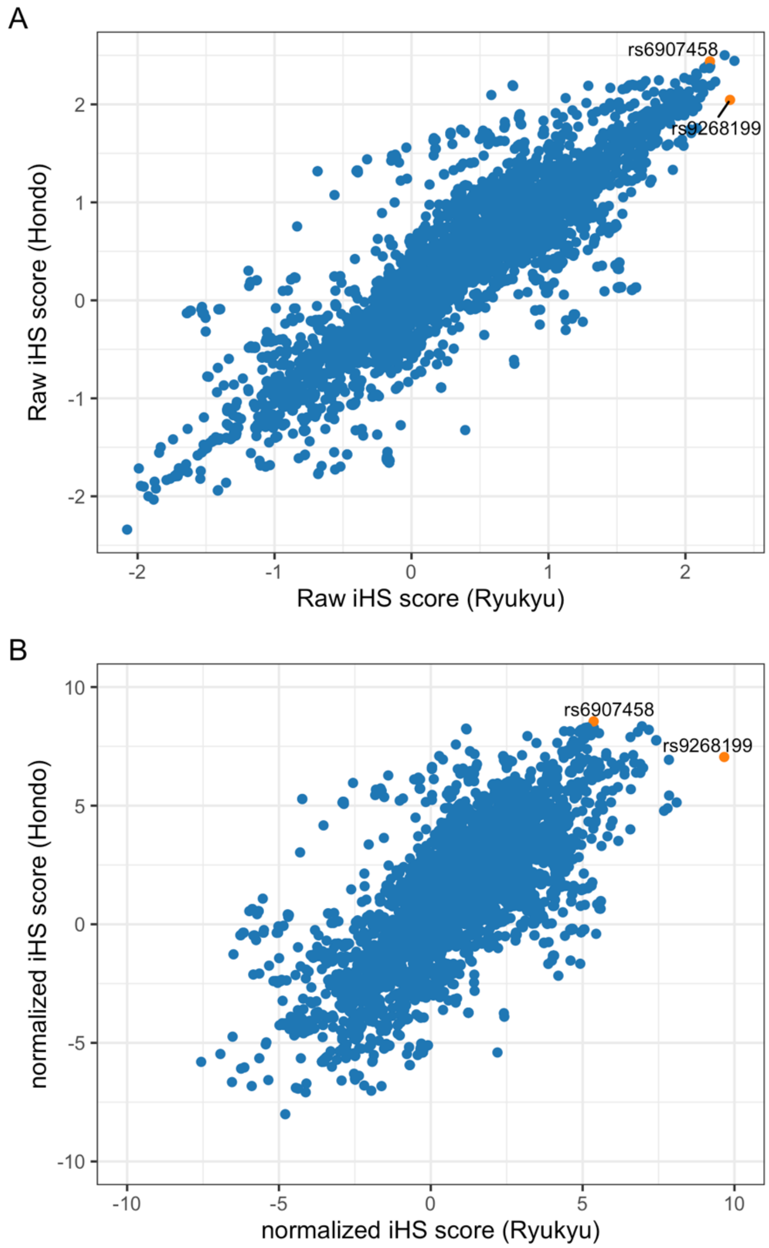


**Supplementary Figure 7 | Comparison of Raw and Normalized iHS Scores for variants in the HLA Region**

A) Raw iHS scores comparison for variants in the HLA region between Ryukyu (x-axis) and Hondo (y-axis). B) Normalized iHS scores comparison for variants in the HLA region between Ryukyu (x-axis) and Hondo (y-axis). The lead variant rs9268199 in Ryukyu and lead variant rs6907458 in Hondo were highlighted in orange color.

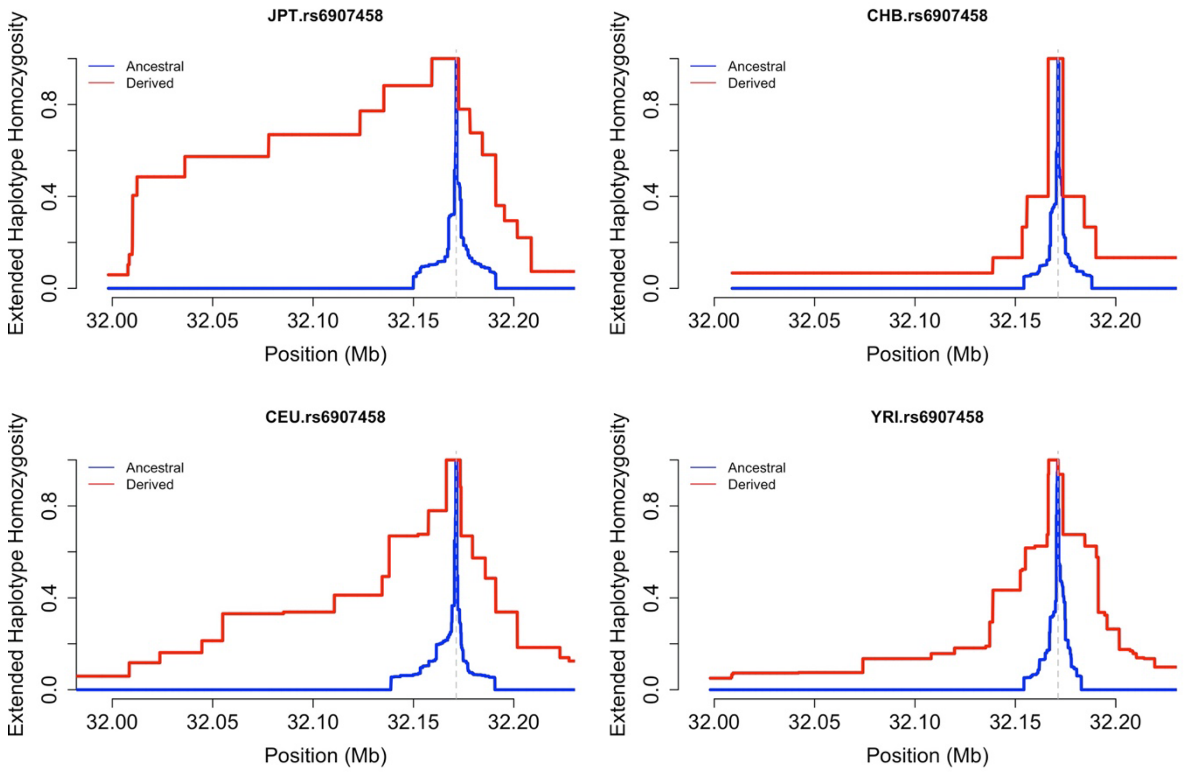


**Supplementary Figure 8 | The Extended Haplotype Homozygosity (EHH) plot of the rs6907458, lead variant suspected to be involved in selection in the MHC region**

The plot was generated for Japanese (JPT), Han Chinese in Beijing (CHB), Utah residents with Northern and Western European ancestry from the CEPH collection (CEU), and Yoruba in Ibadan, Nigeria (YRI) based on the 1000 Genomes Project (1KGP) data.

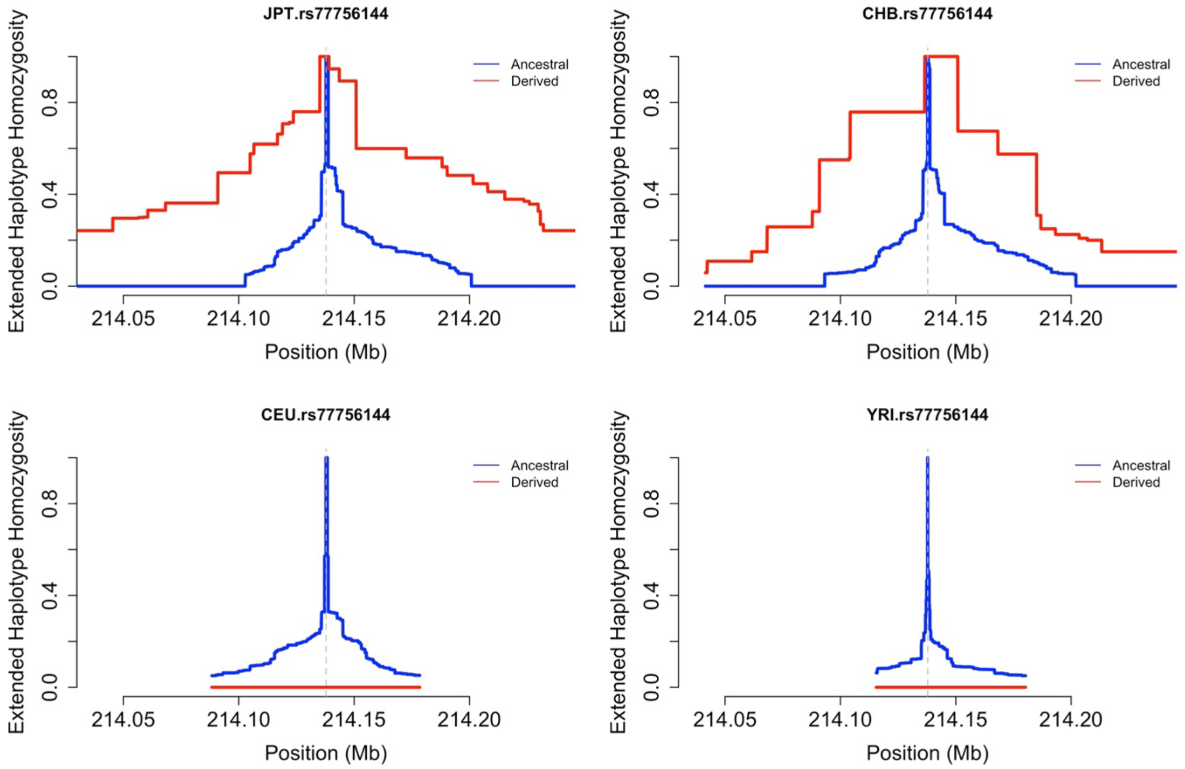


**Supplementary Figure 9 | | The Extended Haplotype Homozygosity (EHH) plot of the rs77756144, lead variant suspected to be involved in selection in the *IKZF2* region**
The plot was generated for Japanese (JPT), Han Chinese in Beijing (CHB), Utah residents with Northern and Western European ancestry from the CEPH collection (CEU), and Yoruba in Ibadan, Nigeria (YRI) based on the 1000 Genomes Project (1KGP) data.

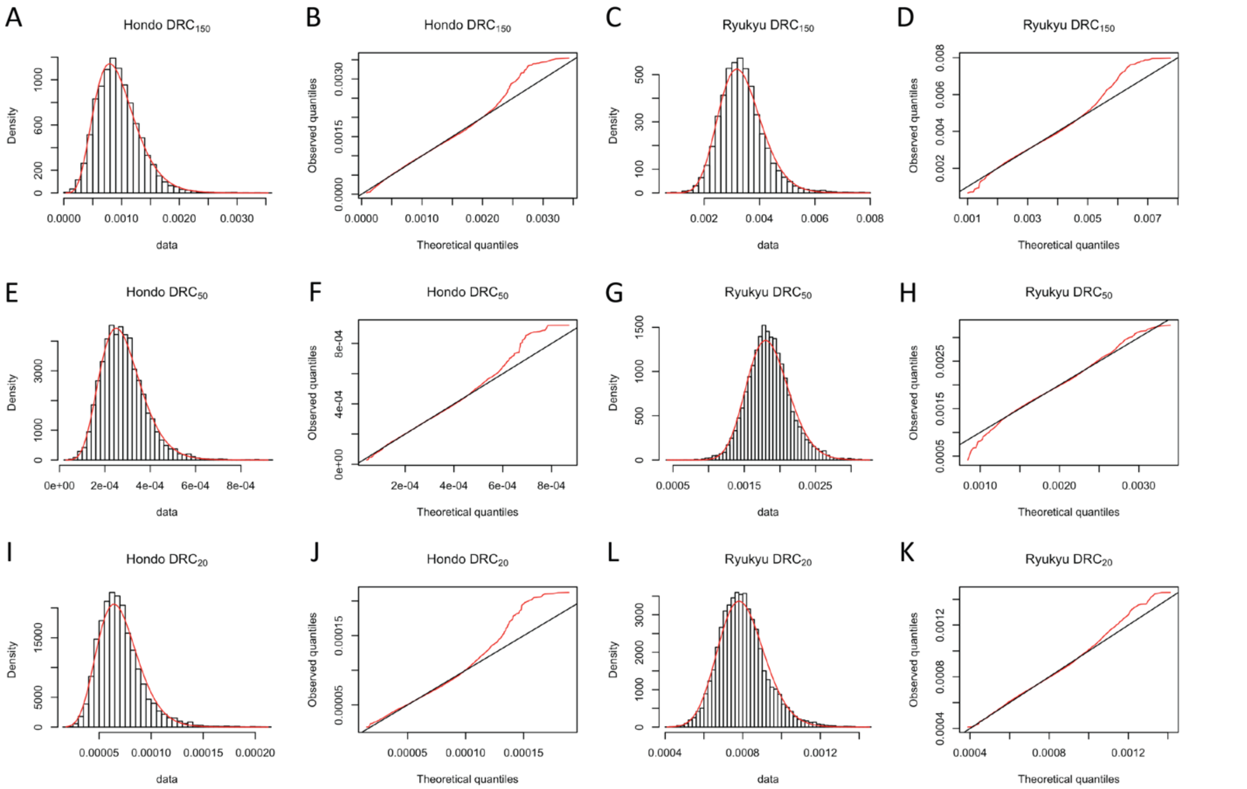


**Supplementary Figure 10 | Empirical null model for the DRC150/50/20 statistic.**
The empirical distribution and Gamma fitting (red curve) for the DRC statistic were examined in the putative neutral regions of the genome (A, E, I for Hondo) and (C, G, L for Ryukyu) across 150, 50, and 20 generations. The quantile-quantile (QQ) plot for the DRC statistic (B, F, L for Hondo) and (D, H, K for Ryukyu) across 150, 50, and 20 generations, red lines indicated observed DRC values.

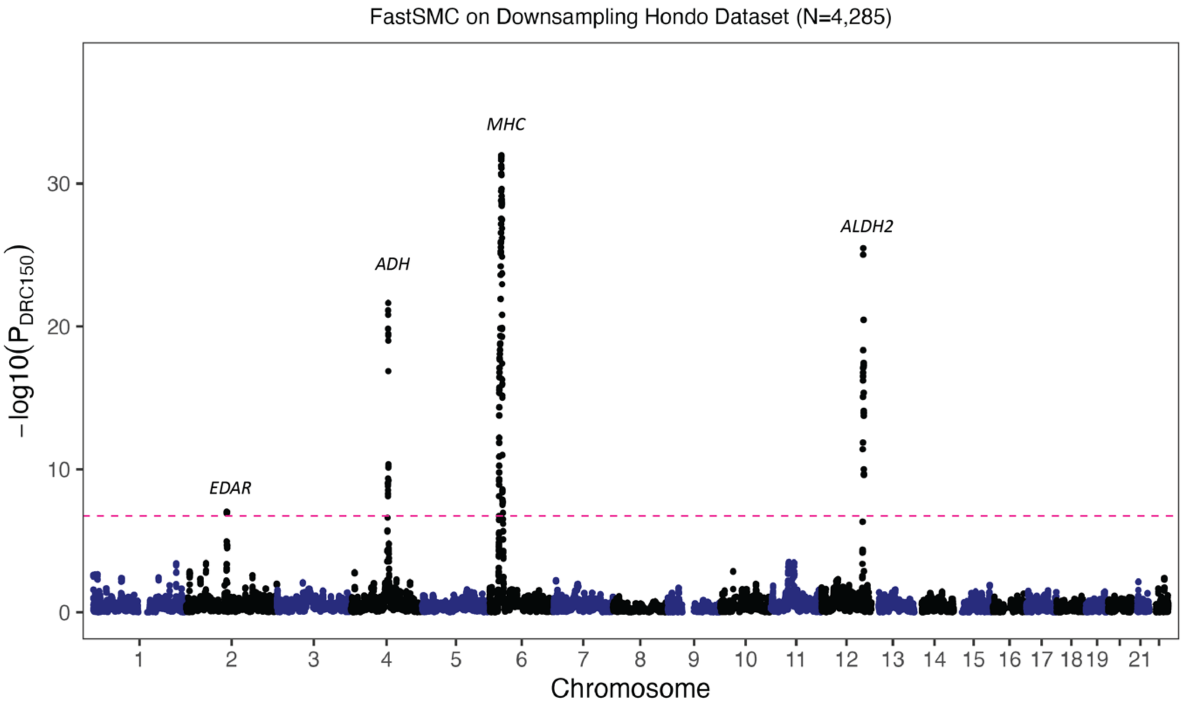


**Supplementary Figure 11 | Genetic loci under positive selection for down-sampled Hondo samples in the past 150 generations based on FastSMC analysis**

The –log10($P_{DRC150}$) value (y-axis) and the chromosomal position (*x*-axis) of each binned region (0.05 cM) are plotted across the genome. The red dashed line indicates the genome-wide significance threshold after Bonferroni correction for the numbers of bins of regions tested ($P < 1.08\times10^{-6}$).

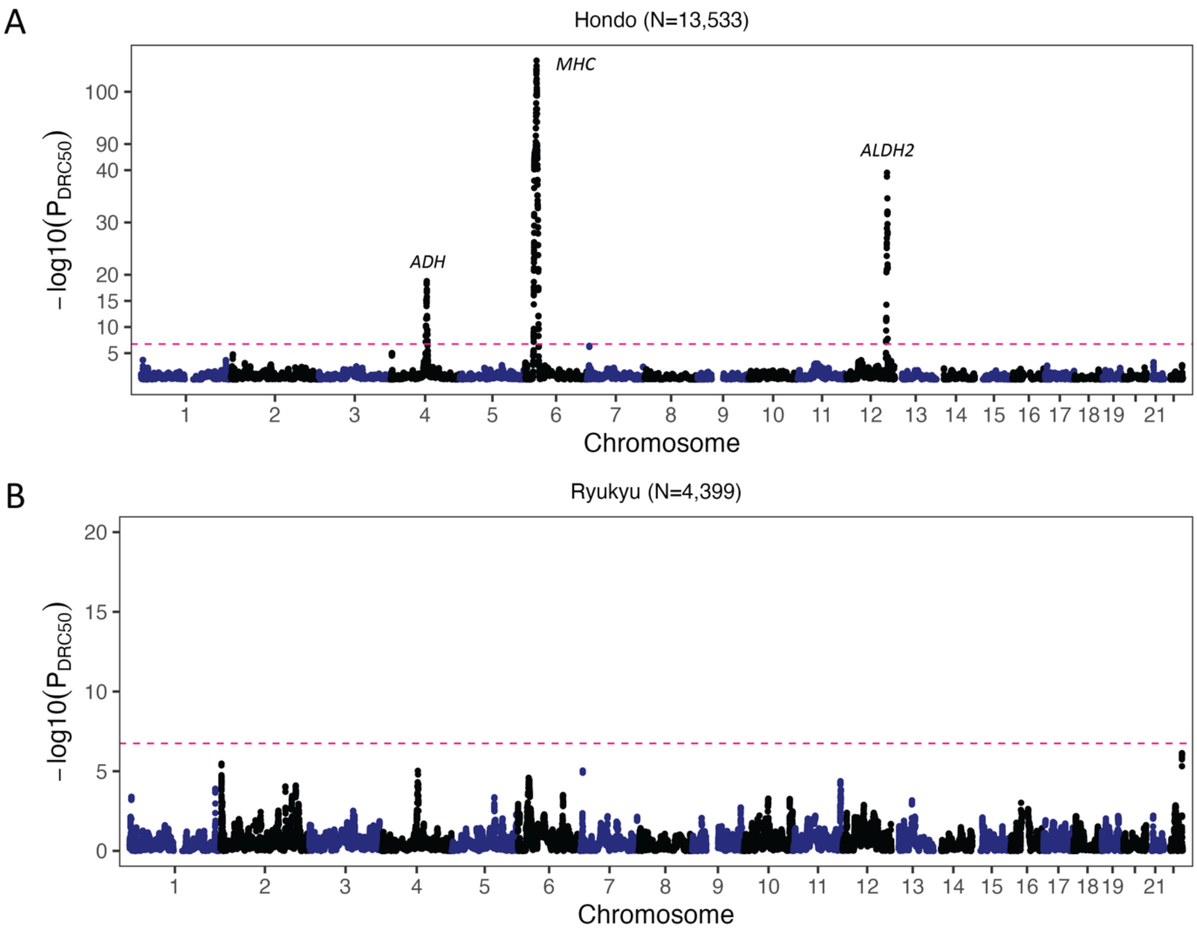


**Supplementary Figure 12 | Candidate loci influenced by positive selection of the Japanese population in the past 50 generations based on FastSMC analysis in Hondo (A) and Ryukyu (B).**

The –log10($P_{DRC50}$) value (y-axis) and the chromosomal position (*x*-axis) of each binned region (0.05 cM) are plotted across the genome. The red dashed line represents the genome-wide significance threshold, which was obtained after Bonferroni correction for the number of bins of regions, subpopulations, and time scale ($P < 1.80\times10^{-7}$). Previously reported gene(s) associated with positive selection are indicated in black

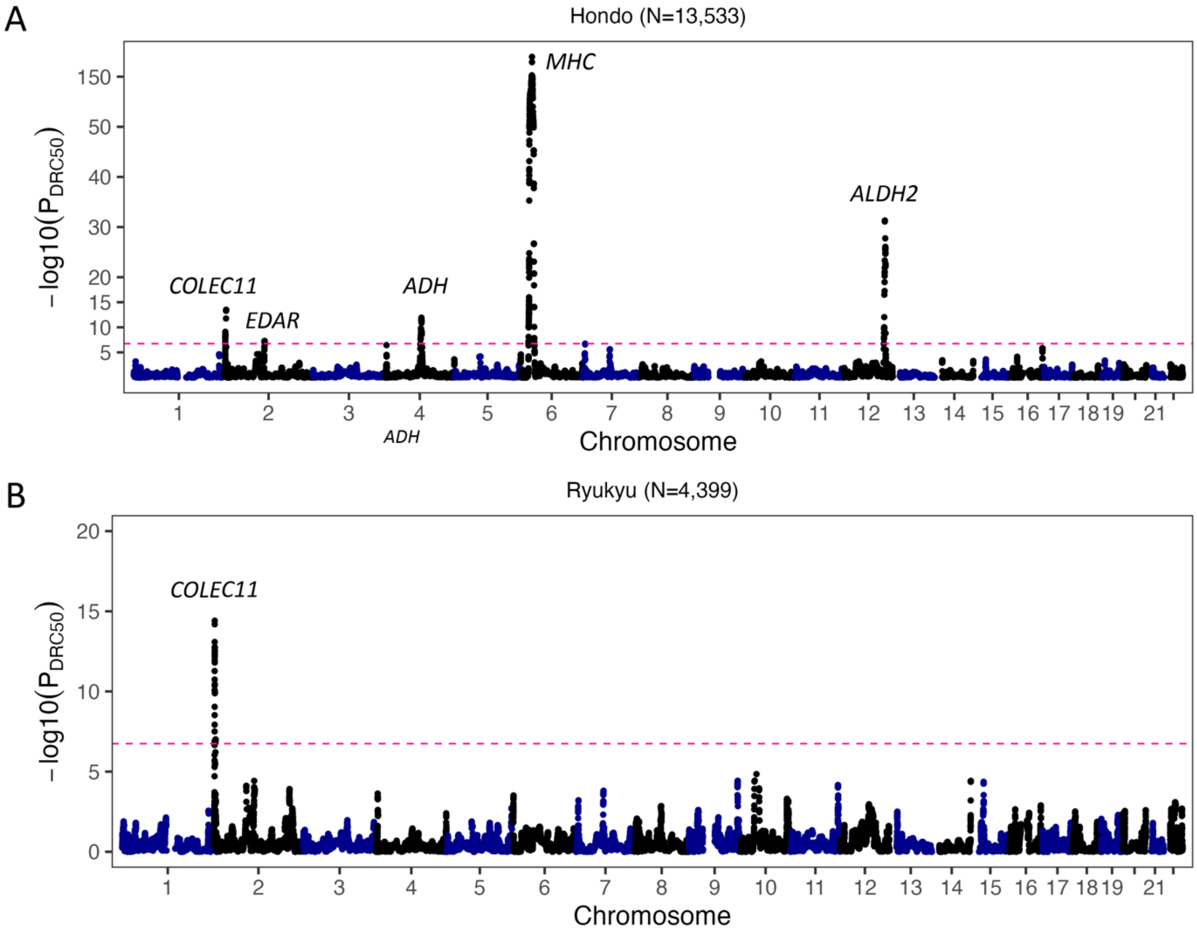


**Supplementary Figure 13 | Candidate loci influenced by positive selection of the Japanese population in the past 20 generations based on FastSMC analysis in Hondo (A) and Ryukyu (B).** The –log10($P_{DRC20}$) value (y-axis) and the chromosomal position (*x*-axis) of each binned region (0.05 cM) are plotted across the genome. The red dashed line represents the genome-wide significance threshold, which was obtained after Bonferroni correction for the number of bins of regions, subpopulations, and time scale ($P < 1.80\times10^{-7}$). Previously reported gene(s) associated with positive selection are indicated in black, while novel genes are highlighted in red.

.

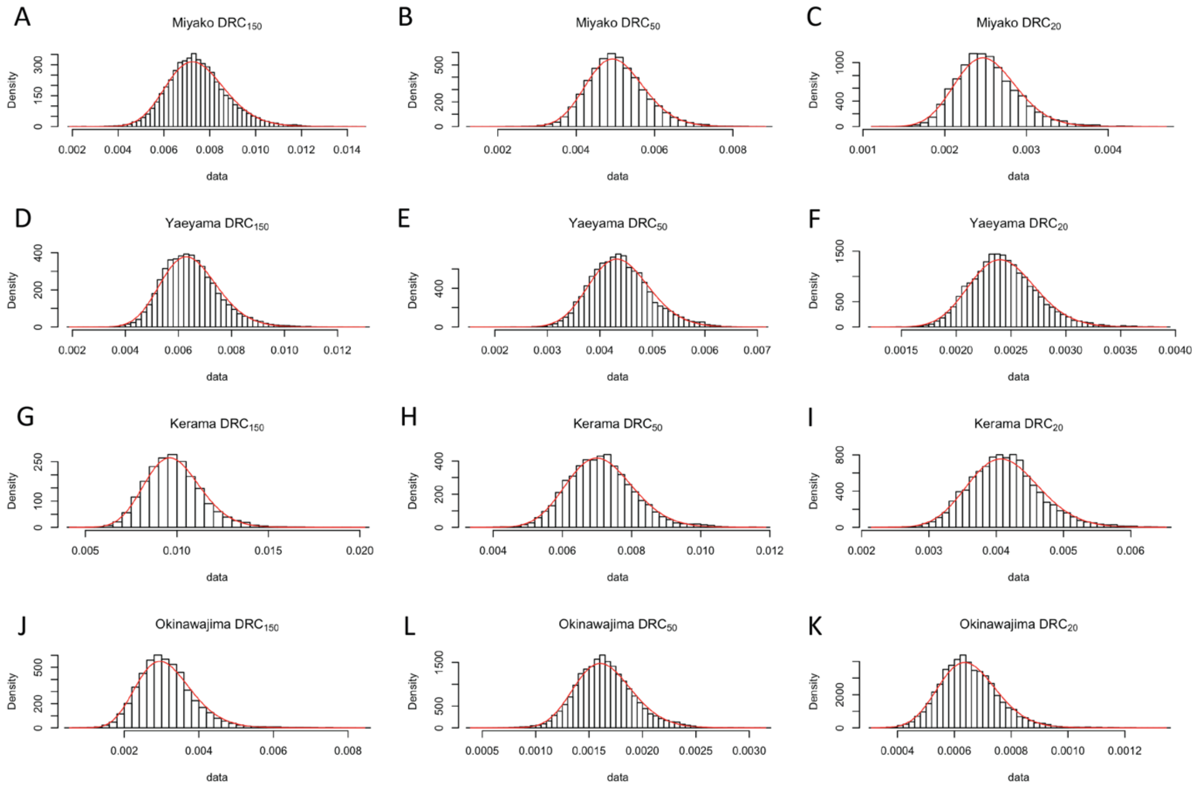


**Supplementary Figure 14 | Empirical distribution of the DRC150/50/20 statistic.**
The empirical distribution and Gamma fitting (red curve) for the DRC statistic were examined in the putative neutral regions of the genome across 150, 50, and 20 generations in 4 Ryukyu subpopulations: A-C representing Miyako, D-F representing Yaeyama, G-I representing Kerama/kume-jima, and J-K representing Okinawa-jima.

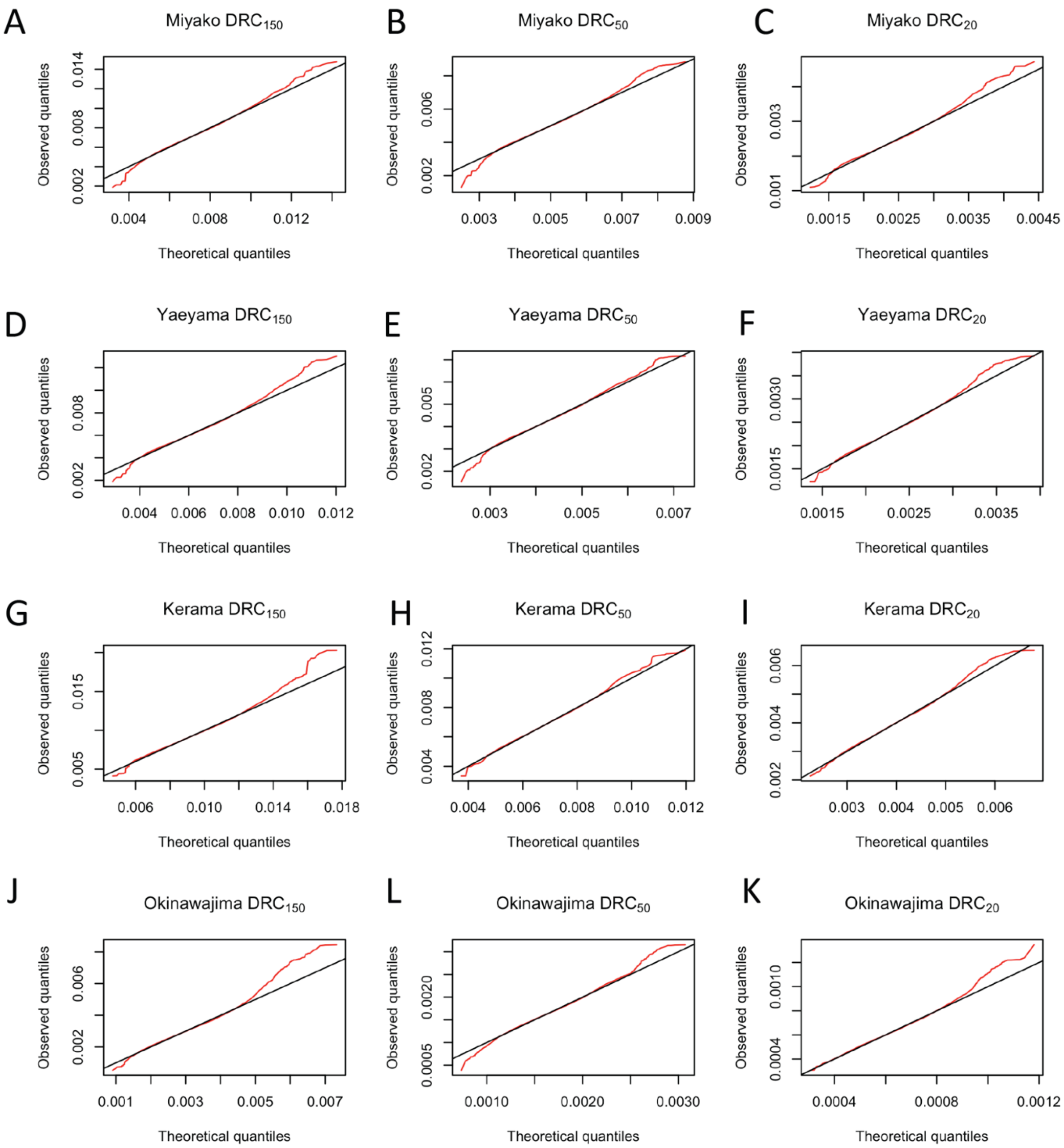


**Supplementary Figure 15 | The quantile-quantile (QQ) plot for DRC150/50/20 statistic.** The quantile-quantile (QQ) plot for the DRC statistic: A-C representing Miyako, D-F representing Yaeyama, G-I representing Kerama, and J-K representing Okinawa-jima. Red lines indicated observed DRC values.

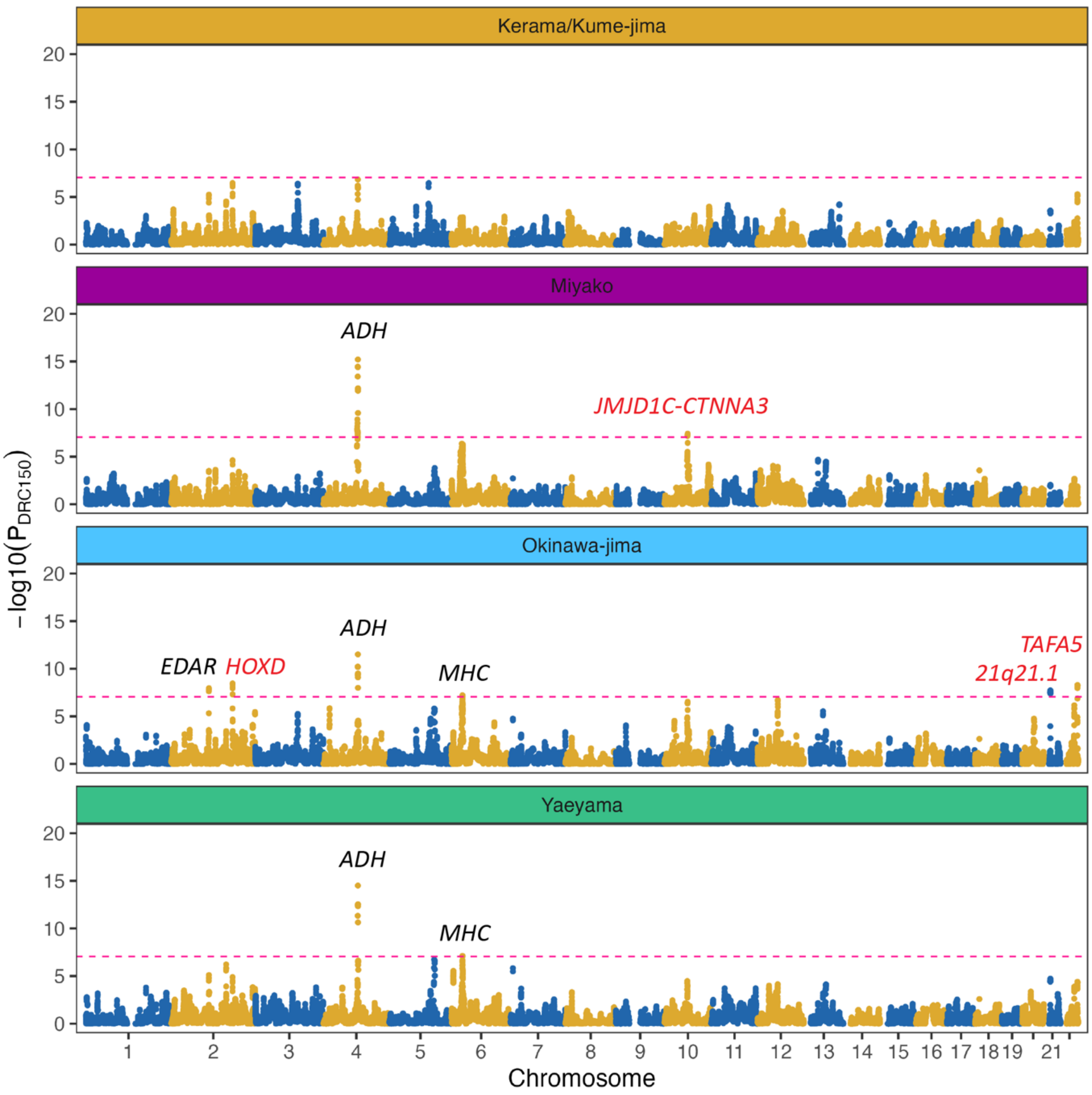


**Supplementary Figure 16 | Genetic loci under positive selection of the Ryukyu subpopulations in the past 150 generations based on FastSMC analysis**

The –log10($P_{DRC150}$) value (y-axis) and the chromosomal position (*x*-axis) of each binned region (0.05 cM) are plotted across the genome. The red dashed line indicates the genome-wide significance threshold after Bonferroni correction for the numbers of bins of regions tested ($P < 9\times10^{-8}$). Candidate targets previously reported to be involved in positive selection in the Japanese population are colored in black, and novel candidates potentially influenced by selection in red.

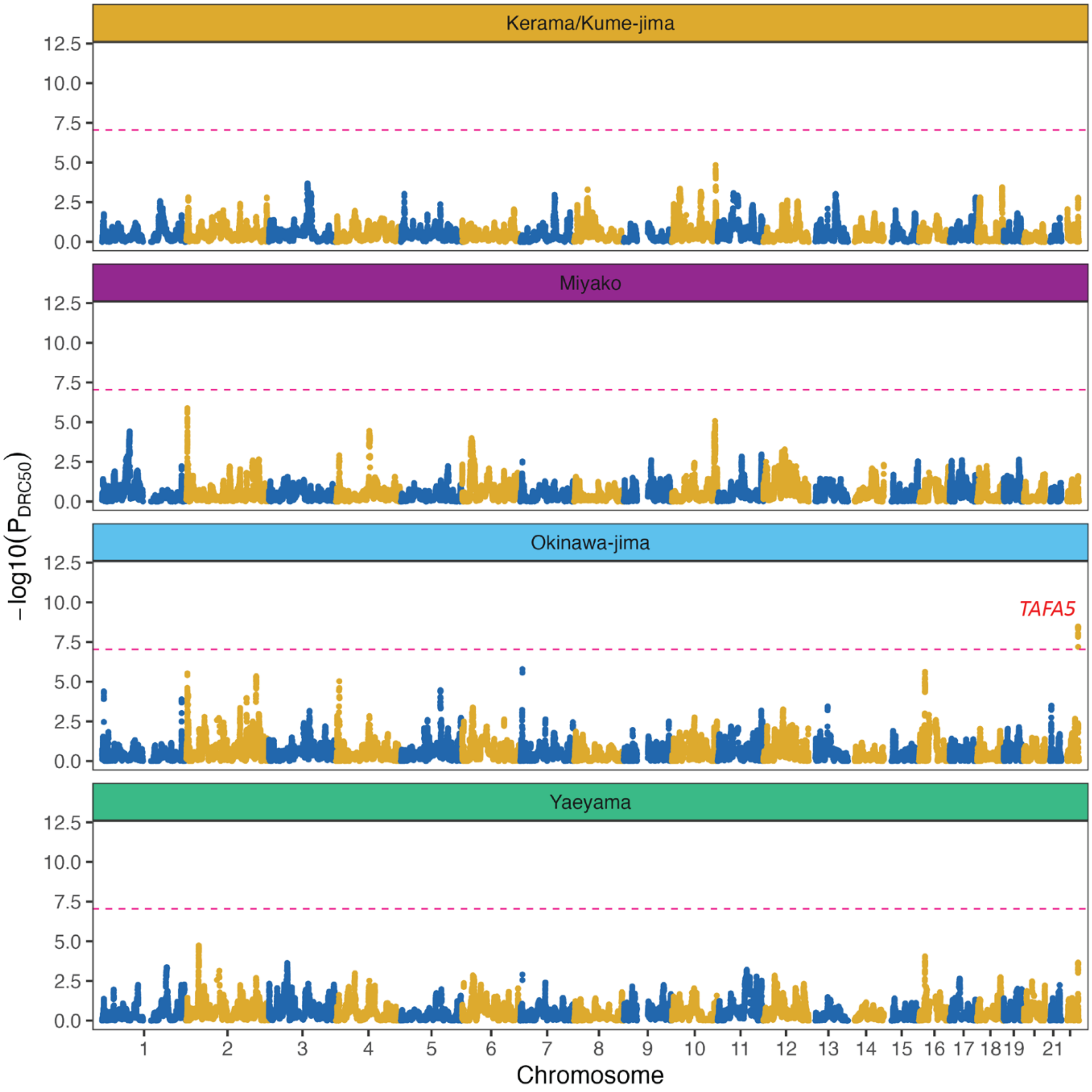


**Supplementary Figure 17 | Genetic loci under positive selection of the Ryukyu subpopulations in the past 50 generations based on FastSMC analysis**

The –log10($P_{DRC50}$) value (y-axis) and the chromosomal position (*x*-axis) of each binned region (0.05 cM) are plotted across the genome. The red dashed line indicates the genome-wide significance threshold after Bonferroni correction for the numbers of bins of regions tested ($P < 9\times10^{-8}$). Gene(s) previously reported to be involved in positive selection in the Japanese population are colored in black, and novel genes in red.

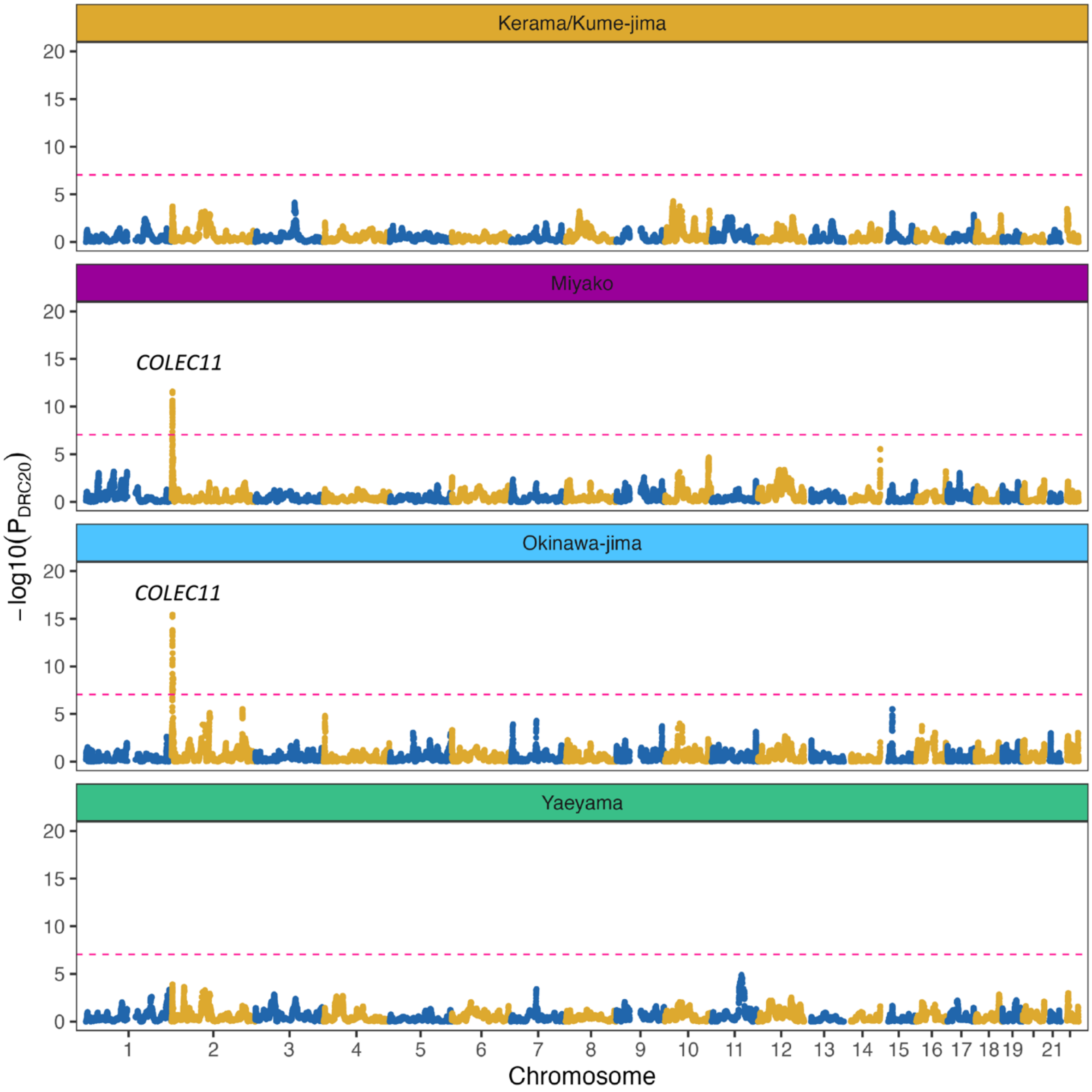


**Supplementary Figure 18 | Genetic loci under positive selection of the Ryukyu subpopulations in the past 20 generations based on FastSMC analysis**
The –log10($P_{DRC20}$) value (y-axis) and the chromosomal position (*x*-axis) of each binned region (0.05 cM) are plotted across the genome. The red dashed line indicates the genome-wide significance threshold after Bonferroni correction for the numbers of bins of regions tested ($P < 9\times10^{-8}$). Gene(s) previously reported to be involved in positive selection in the Japanese population are colored in black, and novel genes in red.

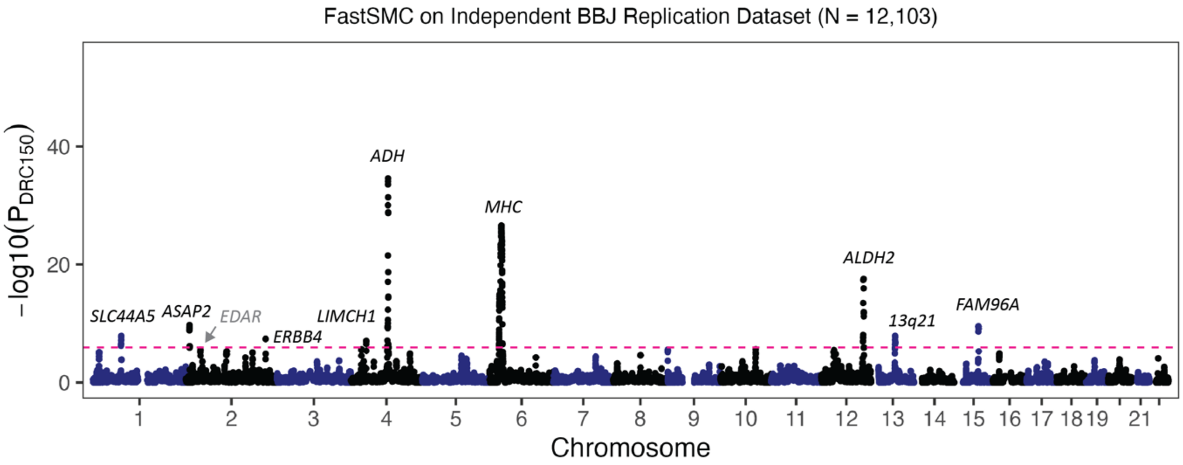


**Supplementary Figure 19 | Genetic loci under positive selection in the past 150 generations based on FastSMC analysis with independent replication samples**

The –log10($P_{DRC150}$) value (y-axis) and the chromosomal position (*x*-axis) of each binned region (0.05 cM) are plotted across the genome. The red dashed line indicates the genome-wide significance threshold after Bonferroni correction for the numbers of bins of regions tested ($P <$ $1.13\times10^{-6}$, 0.05/44,294 bins). We marked signal at *EDAR* in grey as it slightly falls short of the genome-wide significance threshold ($P_{DRC150}$ = $8.03\times10^{-6}$).
